# Communication of information on benefits and harms of multiple competing medical interventions: a three-group, open label randomised controlled trial

**DOI:** 10.1192/bjp.2026.10555

**Published:** 2026-03-25

**Authors:** Edoardo G Ostinelli, Orestis Efthimiou, Adeola Agunbiade, Huseyin Naci, Andrea Cipriani

**Affiliations:** Department of Psychiatry, University of Oxford, Oxford OX3 7JX, UK; Oxford Precision Psychiatry Lab, NIHR Oxford Health Biomedical Research Centre, Oxford OX3 7JX, UK; Oxford Health NHS Foundation Trust, Warneford Hospital, Oxford OX3 7JX, UK; Institute of Primary Health Care (BIHAM), University of Bern, Bern, Switzerland; Abuja, Nigeria; Department of Health Policy, London School of Economics and Political Science, London, UK; Department of Psychiatry, University of Oxford, Oxford OX3 7JX, UK; Oxford Precision Psychiatry Lab, NIHR Oxford Health Biomedical Research Centre, Oxford OX3 7JX, UK; Oxford Health NHS Foundation Trust, Warneford Hospital, Oxford OX3 7JX, UK

## Abstract

**Background:**

Information about a treatment’s benefits and harms to a patient often relies on text. However, for many medical conditions, patients must trade-off benefits and harms across multiple competing treatments. How to appropriately communicate the information on benefits and harms to patients is unknown.

**Aims:**

We compared three communication tools using textual information (Cochrane summary of findings table) or increasing combinations of textual and graphical information (Kilim plot and Vitruvian plot, respectively) to convey the available evidence.

**Method:**

The CICERO (CommunICation of bEnefit Risk information: an Online randomised controlled trial) trial is a three-group, parallel, open label, automated randomised controlled trial (NCT05917639). We recruited participants aged between 18 and 65 years old from the general population. Participants were randomly allocated (1:1:1) to one of the three communication tools providing information on competing fictional treatments for social anxiety and asked to choose one based on externally provided preferences.

The primary outcome was the perceived level of decisional conflict when deciding a treatment (Decisional Conflict Scale, 0=best, 100=worst). As this was an all-or-nothing single-visit trial, only participants contributing with data contributed to the primary analyses (modified intention to treat).

**Results:**

We recruited 2,178 adults between 01-June-2023 and 27-Nov-2023. Vitruvian and Kilim plots outperformed the Cochrane summary of findings table on the primary outcome (adjusted mean difference -10.9; 95%CI [-13.5; -8.2], p<0.0001 and -9.7 [-12.4; -7.1], p<0.0001, respectively). Results varied by participants’ literacy and numeracy skills, lived experience of the condition of interest, ethnic group, sex assigned at birth, and age.

**Conclusion:**

Combining graphical and textual information as opposed to text only improved the communication and reduced the decisional conflict when choosing across multiple competing medical interventions. Organisations involved in disseminating scientific evidence should consider endorsing a combined graphical and textual approach, adopting more intuitive and accessible communication methods. We identified several prognostic factors that should inform the development of future patient decision aids and communication of scientific findings.

**Trial Registration Number:**

NCT05917639.

## Introduction

Effective communication of benefits and harms of medical interventions equips people with the evidence base needed during shared decision-making (SDM) discussions with a healthcare professional.^1,2^ Patient decision aids (PDAs), including evidence communication tools, are valuable instruments before, during, and after SDM in clinical practice. Their effectiveness is affected by how people understand and engage with the format they are delivered in.^2,3^ When multiple competing interventions are available for the medical condition of interest, trade-offs both within each intervention (i.e. between benefits and harms) as well as across interventions must be considered^4^. This complex task may translate into decisional conflict, jeopardising engagement and empowerment in one’s health care.^2,5^

Equity in accessing and understanding information is essential: people accessing medical information have variable levels of numeracy and literacy skills, preferences, and needs^6^. If not adequately supported by accessible and user-friendly communication, people may be at risk of misunderstanding and distrusting the available evidence or may face barriers in accessing optimal levels of care.^2,7,8^ To effectively equip people with the essential tools to make choices about their health, simply providing information is not enough^9^. After the supporting evidence is processed and understood, people should feel confident enough in the acquired knowledge to actively engage in SDM, particularly when the treatment options involve significant harms or other types of disutility.^1,2^ The combination of graphical and textual communication of benefits and harms has been long advocated to adapt and tailor the presentation of information to the preferences, proficiencies, and unique socio-cultural backgrounds of users.^1,7,8^

Among the available communication tools for multiple competing medical interventions, the summary of findings table is widely used by Cochrane and GRADE systematic reviews to communicate evidence to wide audiences, including the lay public, using textual information.^10,11^ Alternatives, such as Kilim plots and Vitruvian plots, combine graphical and textual information to communicate how well treatments work, uncertainties, and availability of evidence.^12,13^ However, it remains unclear whether these more advanced tools offer any added benefit for the effective communication of information on the benefits and harms of multiple interventions.

CICERO is an open-label, three-group, parallel, online automated randomised trial aimed at determining how well the above-mentioned three communication tools, which use different levels of textual and/or graphic languages, conveyed benefits and harms information of multiple competing medical interventions to the general population.

## Method

### Study design and participants

CICERO is a three-group, parallel, open label, automated randomised controlled trial. Eligible participants were adults aged between 18 and 65 years old (inclusive), willing and able to give informed consent for participating in the study, and sufficiently fluent in English. As the trial was online, potentially eligible participants had to have access to internet. There were no country-specific restrictions, and participation in the trial was not restricted to any specific health status (i.e. no definition of presence or absence of a specific medical condition was defined in the eligibility criteria) with no further eligibility criteria to maximise representativeness with real-world population. Information about the trial and the participant information sheet were distributed by several patient-oriented initiatives and charities to their user base via mailing lists, social media, and websites (alphabetical order): Affa Sair (https://www.affasair.org), Bipolar UK (https://www.bipolaruk.org), British Menopause Society (https://thebms.org.uk), Crohn’s & Colitis UK (https://crohnsandcolitis.org.uk), Diabetes UK (https://www.diabetes.org.uk), McPin Foundation (https://mcpin.org), MQ Mental Health (https://www.mqmentalhealth.org), NIHR Be Part of Research (https://bepartofresearch.nihr.ac.uk), Pain Concern (https://painconcern.org.uk), Pain UK (https://painuk.org), Parkinson’s UK (https://www.parkinsons.org.uk), Psoriasis Association (https://www.psoriasis-association.org.uk), Versus Arthritis (https://www.versusarthritis.org), Women’s Health Concern (https://www.womens-health-concern.org). We selected patient-oriented initiatives and charities focusing on conditions for which multiple competing medical interventions are available, either for the condition itself or for their common comorbidities. The authors assert that all procedures contributing to this work comply with the ethical standards of the relevant national and institutional committees on human experimentation and with the Helsinki Declaration of 1975, as revised in 2013. All procedures involving human subjects/patients were approved by the University of Oxford (R86270). The local ethical committee confirmed the opening of the study on 31-May-2023, and we registered the CICERO trial on Clinicaltrials.gov on the same day (31-May-2023). The first participant was recruited on 01-June-2023, in line with the ICMJE timeline definition that registration should occur “at or before the time of first patient enrolment”. Participants were not remunerated for their involvement in the trial.

### Randomisation and masking

An automated, computer-based algorithm randomly assigned eligible participants in real-time (1:1:1) to one communication tool (i.e. summary of findings table, Kilim plot, Vitruvian plot).14 All questionnaires were administered using an online platform and followed a standardised process with no human-to-human interaction throughout the study. The trial consisted of a single online visit (“all-or-nothing” design) starting with the first user-led interaction with the system (informed consent) and ending with the online administration of the questionnaires. Participants could pause the visit at any time and resume their session at their convenience. As blinding was not possible due to the nature of the interventions, participant information sheets did not include specific information regarding the communication tools investigated in the trial to limit bias due to knowledge of which intervention participants were allocated to. Similarly, information sheets did not indicate any of the investigated communication strategies as potentially superior to the others. Informed consent from the potentially eligible participants was received before the delivery of trial procedures.

### Procedures

Eligible participants were asked to complete the baseline assessment, including basic demographic data and an assessment of their health literacy skills (defined as a combination of numeracy and literacy) using a validated questionnaire, the Newest Vital Sign NVS, British version).^15,16^ We also asked participants whether they ever received or sought medical attention and medical treatment for social anxiety disorder (social phobia) to control for the potential impact of personal experiences on the health condition used in the fictional scenario. Participants were provided written instructions to familiarise themselves with the communication tool they had been allocated to. There was no time restriction to complete the trial-related procedures and participants could access the instructions at any time (supplementary pp 8-15). Then, participants were presented with a clinical scenario in which they were asked to impersonate a fictional patient choosing between five different medications for social anxiety after looking at their profiles over five benefit and harm-related outcomes: response, quality of life, acceptability, headache, and nausea. Participants were provided with a given set of preferences on these outcomes and were instructed to carefully consider their trade-offs (p 20). The data on the five outcomes for the five medications and a reference (placebo) were identical across the groups but were differently communicated as per the allocated communication tool. At the end, participants were asked to answer several questions to evaluate the performance of the communication tool, as well as the choice they would have made with their own set of preferences.

For the fictional scenario, we chose social anxiety as it is a common condition in the general population. The condition often starts during the teenage years – but can emerge at any age.^17^ Although cognitive behavioural therapy (CBT) is generally considered the best treatment for social anxiety we did not include it in our scenario. The reason is that our goal was to evaluate multiple competing treatments for which enough information is available on their overall benefits and harms, while harms associated with psychological therapies remain under-explored and under-reported.^18,19^ Additionally, CBT may be less known and intuitively understood compared to “medications” (we purposedly did not emphasise a specific treatment or treatment class, e.g., we avoided using terms like selective serotonin reuptake inhibitors or escitalopram).^17^

### Interventions

The three interventions differed in whether the various domains related to the communication of benefits and harms information were conveyed using graphical and/or textual information (box 1). An overview of the administered interventions is provided in the extended data, with further details in the supplementary information (pp 16-21).

The Cochrane summary of findings table communicates benefit-harm information in a comprehensive format, mainly using textual communication. It is intended for a broad audience, including end-users of systematic reviews and guidelines. The use of summary of findings tables is recommended by Cochrane and they are included in most of their published systematic reviews. We focused on the following columns of the summary of findings template: outcomes, illustrative comparative risks, relative effects (95% confidence interval, CI), and number of participants and studies.^10^

The Kilim plot conveys benefit-harm information in a tabular format, using a mix of textual and graphical elements. All evidence is combined in a single table, facilitating comparison between treatments across multiple outcomes.^12^

The Vitruvian plot communicates benefit-harm information in both textual and graphical format. The evidence from all outcomes is condensed in a single-coloured plot per treatment, allowing to gauge its overall profile and explore trade-offs within and across treatments.^13^

### Measures

The communication of information about benefits and harms involves three key steps: firstly, understanding the available information; secondly, feeling confident about one’s own understanding; and finally, feeling prepared to actively participate in SDM. Additional details are provided in the supplementary information (pp 23-44).

#### Decisional Conflict Scale, low literacy version (primary outcome)

The Decisional Conflict Scale (DCS) measures perceptions of difficulty in making decisions, including lack of information to help choose between options and feeling unclear about one’s own preferences. We used DCS as an overall measure to evaluate the perceived effectiveness of the communication of information process. DCS can be further organised into four sub-scales: feeling uninformed (knowledge), feeling uncertain about best choice (certainty), feeling unclear about personal values for benefits and harms (values clarity), feeling unsupported in decision-making (support).

#### Information comprehension (secondary outcome)

After participants were presented with a short fictional clinical scenario, they were asked to indicate which medication they would opt for. One out of five possible answers had been a-priori defined as correct during trial development and had been previously tested with five members of the public (supplementary information, p 22).^20,21^ We also explored frequency of wrong answers: “medication 1”, which prompted participants to consider the certainty of effects between multiple outcomes, “medication 3”, which prompted participants to jointly consider magnitude, direction, and certainty of effects, “medication 4”, which had missing information for one relevant outcome (availability of evidence), “medication 5”, which prompted participants to consider direction and magnitude of effects, and hesitancy (“I don’t know”).

#### Decision Self-Efficacy scale (secondary outcome)

The Decision Self-Efficacy (DSE) scale measures self-confidence and belief in one’s ability to make decisions about their own health.

#### Preparation for Decision Making scale (secondary outcome)

The Preparation for Decision Making (PDM) scale measures a user’s perception of how useful a decision support tool is in preparing them to talk to a health professional about a health decision. The original scale has 10 items, but a single item (“Help you recognise that a decision needs to be made?”) was omitted as it was considered not relevant to this study, as done by Hopkin and colleagues.^22^

#### Time to completion (secondary outcome)

We measured the time each participant required to familiarise themselves with the intervention (starting time: administration of the intervention) and complete the questionnaires (ending time: completion of all questionnaires). The overall time spent was considered a proxy measure of feasibility.

#### Drop-out rate (additional analysis)

We measured (binary) discontinuation from the intervention after being allocated to an arm of interest (post-hoc analysis).

#### Associations between outcomes

We estimated the association between outcomes throughout the chain of communication of information on benefits and harms: first, participants needed to understand the available information, then to rate how confident they felt about the available information, and finally how these prompted having an active role into SDM.

### Statistical analysis

Information about sample size and power is provided in the supplementary information (p 4). The primary analyses were conducted following a modified intention-to-treat approach for all the outcomes as described by the risk of bias 2 guidance, as this was a simultaneous trial (all-or-nothing adherence): those who did not initiate the unique study visit were excluded from the analyses.23 Relative effects between interventions were evaluated using a linear regression model for continuous outcomes and a logistic regression model for binary outcomes, using age, sex assigned at birth, ethnic group (using the United Kingdom Office for National Statistics macro-categories, see supplementary information p 6), literacy and numeracy, education, and exposure to social anxiety as covariates. Relative intervention effects were expressed as adjusted mean difference (aMD) and adjusted odds ratio (aOR). To explore the association across outcomes, we estimated the Pearson correlation (ρ) between the considered outcomes, and the difference in rating questionnaires across participants that correctly understood the information and those who did not. Additional information on models definition is provided in the supplementary information (pp 23-61). All analyses were performed in R (version 4.3.1).

## Results

### Participant characteristics

**Fig 1:**
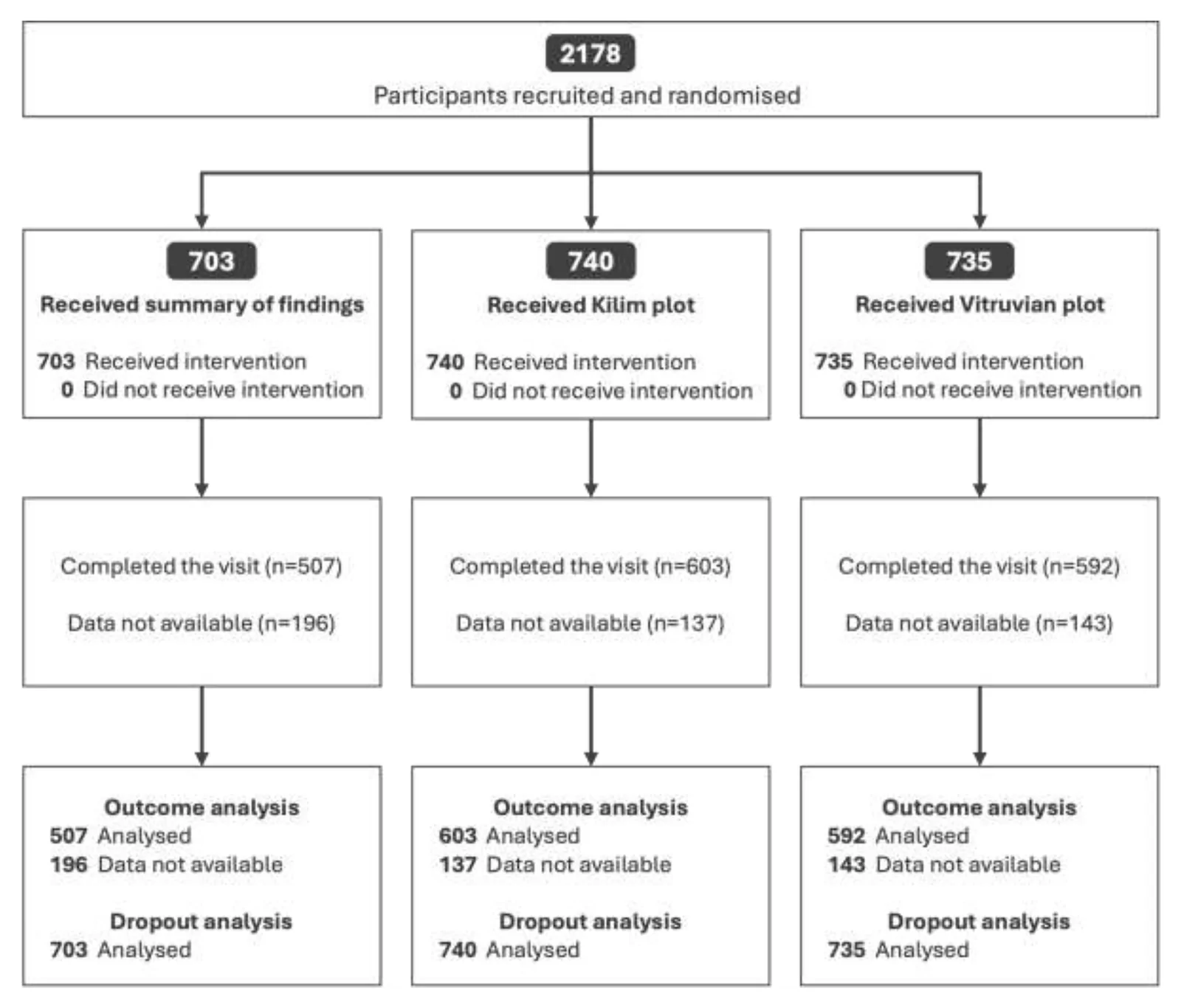
Flow of participants through study. As the automated trial consisted of a single online visit (all-or-nothing) participants that did not complete the study visit could not contribute data to the outcome analyses.

We randomly assigned 2,178 participants: 703 (32.3%), 740 (34.0%), and 735 (33.7%) to the summary of findings, Kilim plot, and Vitruvian plot groups, respectively (Fig 1).

The mean age of the sample was 48.5±15.4 years old, with 67% of the participants (n = 1,137) identifying as female (table 1). Overall, 34% (n = 582) reported their highest degree to be lower than university, 92% (n = 1,562) identified themselves as “white”, and 20% (n = 339) reported NVS scores suggestive of limited literacy and numeracy (table 1). Overall, 476 participants (22%) did not complete the trial procedures after providing informed consent, of which 196 (28%) in the summary of findings group, 137 (19%) in the Kilim plot group and 143 (19%) in the Vitruvian plot group (Fig 1).

### Relative effects between interventions

#### Decisional Conflict Scale, low literacy version

The Kilim plot (mean=21.9, SD=22.0; adjusted mean difference versus summary of findings -9.7, 95% confidence interval -12.4 to -7.1, p=6×10^-13^) and the Vitruvian plot (mean=20.9, SD=21.6; adjusted mean difference -10.9, -13.5 to -8.2, p=2×10^-15^) groups were associated with lower decisional conflict compared to the summary of findings group (mean=32.1, SD=24.5). There was no strong evidence of differential effects between participants allocated to the Vitruvian plot and those in the Kilim plot group (aMD=-1.1, -3.7 to 1.4], p=0.38) (table 2).

Factors contributing to improved performances at the DCS (i.e. prognostic factors) were increasing age (regression coefficient per year of age -0.24, -0.3 to -0.2, p=2–10-9) and increasing literacy and numeracy (regression coefficient per point increase -1.4, -2.4 to - 0.5, p=0.003). Self-identification with a black ethnic group (coefficient versus white = 15.8, 3.7 to 27.9, p=0.01) and familiarity with the medical condition used in the clinical scenario (coefficient versus no familiarity 3.0, 0.02 to 5.9, p=0.05) were associated with lower performances (supplementary information pp 24-25). For the remaining factors, this study found no evidence of an association. We found similar findings regarding the performance of the interventions when focusing on the four sub-scales (supplementary information pp 26-33).

#### Information comprehension

**Fig 2:**
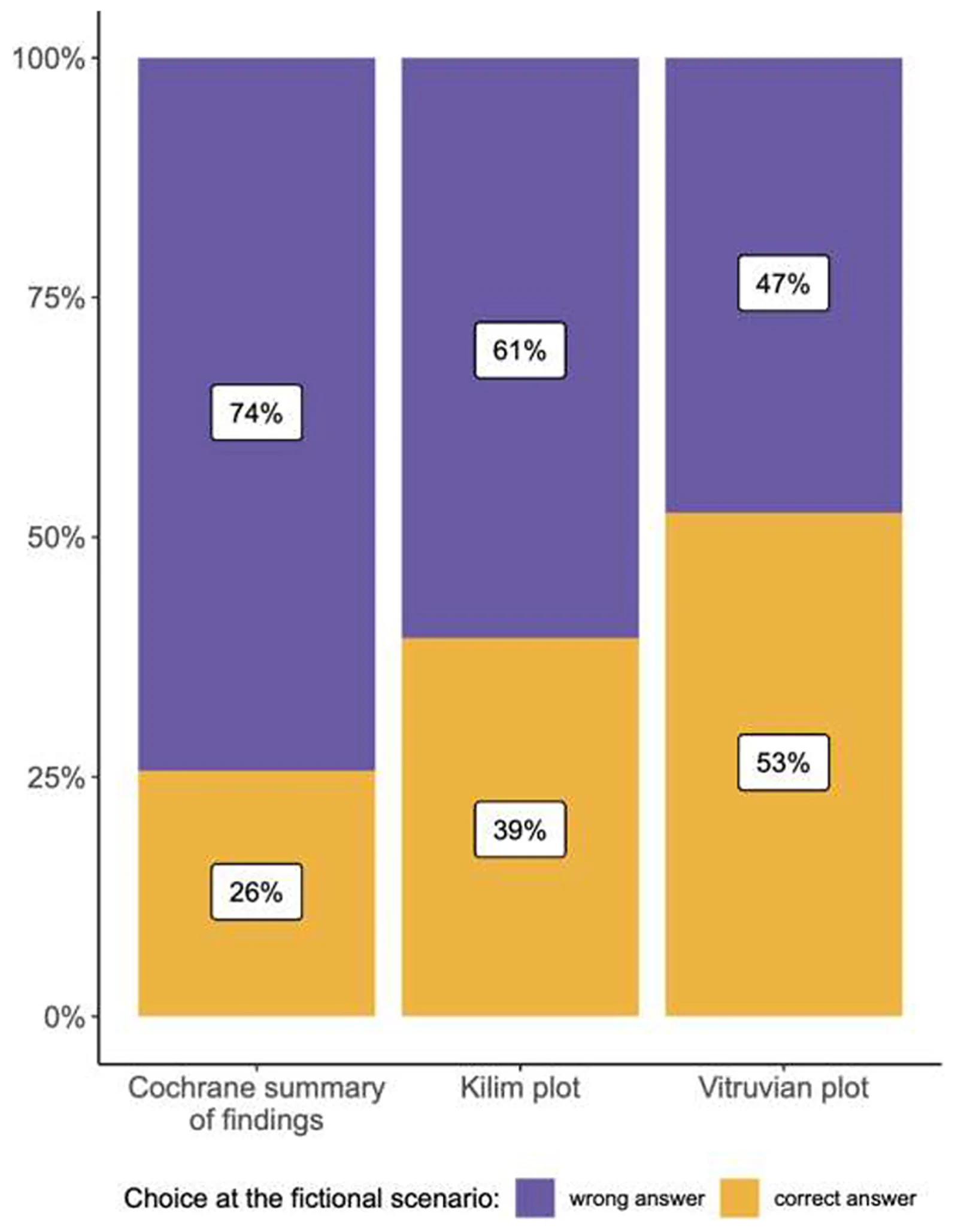
Scenario-based choice with given set of preferences and values. Participants were asked to select one out of five possible medications or say they did not know what to answer. Based on the provided scenario and the given set of preferences and values, only one answer was a-priori considered correct.

26% of the participants (130 out of 507, table 2) allocated to the summary of findings group chose the correct medication, as opposed to 39% in the Kilim plot group (238 out of 603; aOR versus summary of findings 1.85, 1.42 to 2.41, p=5×10^-6^) and 53% in the Vitruvian plot group (311 out of 592; aOR versus summary of findings 3.47, 2.66 to 4.53, p<2×10^-16^; aOR versus Kilim plot 1.88, 1.48 to 2.38, p=2×10^-7^) (Fig 2). Among other prognostic factors, male sex (aOR 1.32, 1.05 to 1.63, p=0.02) was associated with higher odds of answering correctly (further details in supplementary information, pp 37-38, with an overview of identified prognostic factors at pp 50-51).

We found similar rates across the three interventions in terms of participants selecting “medication 1” (certainty of effects between multiple outcomes) and “medication 3” (joint appraisal of magnitude, direction, and certainty of effects) as the answer to the fictional scenario. Missing evidence was more commonly overlooked by participants in both the summary of findings (21%, 106 out of 507) and Kilim plot (18%, 109 out of 603) as opposed to those allocated to the Vitruvian plot (4%, 22 out of 592). Similar findings were observed in terms of participants overlooking direction of effects (summary of findings: 22%, 111 out of 507; Kilim plot: 20%, 123 out of 603; Vitruvian plot: 15%, 89 out of 592). Fewer participants allocated to the Kilim plot (1%, 9 out of 603) and the Vitruvian plot (3%, 17 out of 592) were hesitant to make a decision compared to the summary of findings (8%, 42 out of 507) (supplementary information, pp 34-38).

#### Decision Self-Efficacy scale

Participants allocated to the Kilim plot (mean=75.0, SD=23.3; adjusted mean difference 7.8, 5.0 to 10.6, p=5×10^-8^) and the Vitruvian plot (mean=74.1, SD=23.7; adjusted mean difference 7.4, 4.6 to 10.2, p=3×10^-7^) performed better than those in the Summary of findings group (mean=66.4, SD=25.7) at the DSE, with negligible differences between the first two treatment groups (aMD Vitruvian versus Kilim plot: -0.40, -3.1; 2.3, p=0.77) (table 2). Further details on the prognostic factors are provided in the supplementary information (pp 40-41).

#### Preparation for Decision-Making scale

Participants in the Kilim plot (mean=71.8, SD=23.9; aMD=12.3, 9.3 to 15.3, p=2×10^-15^) and the Vitruvian plot (mean=68.3, SD=25.4; aMD=8.7, 5.7 to 11.7, p=2×10^-8^) groups performed better than the Summary of findings group (mean=59.2, SD=27.1) at the PDM, with participants allocated to the Kilim plot group feeling more prepared for decision-making compared to those having accessed the Vitruvian plot (aMD=-3.6, -6.5 to -0.7, p=0.015) (table 2). Further details on the prognostic factors are provided in the supplementary information (pp 43-44).

#### Time to completion

Participants allocated to the summary of findings table required more time (in minutes) to complete the questionnaire (mean=18.4, SD=9.1) compared to those in the Kilim plot group (mean=15.2, SD=7.5, aMD=-16.4, -20.8 to -11.9, p=7×10^-13^) and in the Vitruvian plot group (mean=17.0, SD=8.9, aMD=-19.4, -23.8 to -14.9, p<2×10^-16^). We did not observe important differences between the Kilim and the Vitruvian plots (aMD=-3.0, -7.3 to 1.3, p=0.2). We found similar findings when retaining the outliers (supplementary information pp 46-47).

#### Choice with participants’ own set of preferences

When looking at the participant choice based on their own set of preferences and values, participants in the Kilim (aOR=4.25, 2.63 to 7.10, p=1×10^-8^) and Vitruvian plot groups (aOR=4.35, 2.69 to 7.27, p=6×10^-9^) had lower odds of being unsure compared to people allocated to the summary of findings table, with the comparison between Kilim and Vitruvian plots showing no evidence of difference (aOR=1.02, 0.56 to 1.87, p=0.9). Further details on the prognostic factors are provided in the supplementary information (p 48).

#### Drop-out rate (additional analysis)

In a post-hoc analysis, participants allocated to the Kilim (aOR=0.59, 0.46 to 0.75, p=3×10^-5^) and Vitruvian plots (aOR=0.62, 0.49 to 0.80, p=0.0002) had lower odds of discontinuing the intervention compared to people in the summary of findings group, with the comparison between Kilim and Vitruvian plots showing no evidence of an effect (aOR=1.06, 0.82 to 1.38, p=0.7) (supplementary information p 49).

#### Associations between outcomes

Overall, better self-efficacy scores positively correlated with improved preparedness to decision-making (ρ=0.63, 0.60 to 0.66, p<0.001) and lower decisional conflict (ρ=-0.60, -0.63 to -0.57, p<0.001); similarly, higher preparedness to decision-making scores correlated with lower decisional conflict scores (ρ=-0.61, -0.64 to -0.58, p<0.001). We observed similar findings across the three interventions (supplementary information pp 53-54). When we removed outliers, we found that time positively correlated with improved performance in terms of self-efficacy, preparedness for decision-making, and decisional conflict for participants allocated to summary of findings but not for Kilim and Vitruvian plot groups (supplementary information pp 55-56).

Participants who correctly understood the available information reported higher decision-self-efficacy (aMD=4.3, 2.0 to 6.7, p=0.0004) and preparedness for decision-making scores (aMD=4.7 2.2 to 7.2, p=0.0002), and lower decisional conflict (aMD=-3.2, -5.4 to -0.9, p=0.006) (supplementary information pp 57-60).

## Discussion

This online randomised trial shows that, when comparing three communication tools of benefits and harms of multiple medical interventions in 2,178 adults from the general population, combined graphical and textual communication (Kilim and Vitruvian plots) was associated with lower decisional conflict in making health-related decisions compared to the textual-only information (summary of findings table). This was particularly evident in the first step of communication, with the Vitruvian plot leading to better information comprehension than the Kilim plot, and both outperforming the summary of findings table. The same effect was observed across subsequent steps, with people receiving a combination of graphical and textual information feeling more confident about it and more prepared for having an active role in SDM. Individual literacy and numeracy skills, prior exposure to the medical condition and treatment of interest, ethnic group, sex assigned at birth, and age emerged as important prognostic factors materially impacting individuals’ potential engagement with SDM.

Our findings suggest that well-designed communication strategies can support people in making an informed decision on multiple medical interventions while allowing them to weigh the corresponding benefits and harms.^24^ This is in line with earlier evidence on the efficacy of tabular data for single medical treatments in the general population.^20,21^ In several medical areas, multiple medical treatments with competing performances are available:^25,26^ the decision on whether to start treatment or not, and which treatment to opt for, relies on subjective preferences and values. In our study, combined graphical and textual information provided advantages over text-only information, with the Kilim plot (39%) performing better than the summary of findings table (26%) in terms of information comprehension, and the Vitruvian plot (53%) outperforming both. While these figures may appear relatively modest in absolute terms, they should be interpreted in the context of our study design: participants were asked to engage with one hypothetical scenario, without the personal relevance or emotional investment typically present in real clinical encounters or if dealing with treatment options that directly affect their own health/condition. Moreover, we intentionally designed the task to be cognitively demanding by including five treatment alternatives to test the limits of comprehension in a complex decision-making context. The entire online task (from providing baseline information to the completion of the last questionnaire) required an active engagement of about 15 to 20 minutes, on average, which is considerably higher to what happens as it involved several research steps (e.g., collection of baseline and outcome information). These results underscore the challenges of promoting patient understanding through standalone materials and highlight the importance of simplifying information, reducing cognitive load, and integrating clinician support to facilitate effective SDM when developing PDAs.

Correctly understanding medical information was associated with feeling more confident about it, feeling more prepared to engage with SDM, and to an overall lower decisional conflict. These domains represent key milestones in the sequence of events that lead to informed decision-making. Challenges in the process of interpreting medical information may translate into barriers in actively making decisions about one own’s health and engaging with healthcare providers.^2^ For instance, not feeling confident may result in disengagement from SDM and subsequently to missed opportunities to access better health care. Feeling confident and ready to engage in SDM might be jeopardised by an underlying misunderstanding of the available information. Combined graphical and textual communication and inclusion of uncertainty and availability regarding the relevant evidence can reduce these challenges and support patients and clinicians throughout the SDM process.^2^ Development of future PDAs should allow for interactive exploration of medical information tailored to the individual needs and preferences,^22^ with digital interventions representing only one of the ingredients for productive and informed SDM.^2,5^

This study found that increasing levels of literacy and numeracy skills led to higher chances of correctly understanding the provided information, feeling confident about it, and experiencing overall lower decisional conflict. This adds to the previously reported positive impact of literacy and numeracy skills on information comprehension.^27^ Visualisation relying on graphical communication may help in accommodating variable levels of numeracy skills from individuals accessing medical information. Increasing attention has been advocated to initiatives promoting and supporting health literacy at an individual level,^28^ but these changes should be accompanied by reforms of the wider societal and structural architecture.^29,30^

An interesting additional finding is the identification of prior exposure to the medical condition and treatment of interest, ethnic group, sex assigned at birth, and age as non-modifiable characteristics that impacted communication of benefits and harms of medical interventions. Our results support recent calls voicing concerns on the current state of diversity and inclusivity related to accessing digital health, particularly with regards to women from minority ethnic groups.^31,32^ The surge of ethnic minority health apps mirrors the need to culturally tailor PDAs and other e-products to specific ethnical communities.^33^ Teams involved in digital health care should include people with lived experience and be ethnically-, gender-, and age-diverse to dismantle inequalities, promote use of digital interventions, and offer trust-building equitable approaches to the community.^2,34,35^ An example is the “Innovating for all” workstream from the UK NHS Race and Health Observatory, focusing on the development and deployment of digital interventions with a particular interest on genomics and precision medicine.^35^ At the same time, it is important to note how these initiatives should be streamlined and part of comprehensive and systemic responses to health inequalities.^35^

Our findings have important implications for various entities. Firstly, organisations involved in the production and dissemination of scientific evidence, such as journals and working groups like Cochrane, GRADE, can leverage these findings to improve their communication of evidence syntheses. Secondly, researchers and practitioners who are developing PDAs can utilise a combination of textual and graphical presentation to promote more effective participation in SDM. Lastly, committees tasked with formulating research reporting guidelines can use our findings to improve their standards.

## Limitations of the study

Our findings may not apply to everyone, as access to digital devices was required given the online nature of the CICERO trial. Although digital access has sensibly improved up to 93% of the UK households after the COVID-19 pandemic, people aged above 65 years old and lower-income households stand at higher risks of digital divide.^36^ When generalising our findings, it is essential to recognise that not all people may want an active role in decision-making nor would like to take part in it.^2^ Our recruited sample showed that one in five had inadequate literacy or numeracy, a finding aligned with the available data on proficiency and education in the United Kingdom.^37,38^ However, it is important to mention that people identifying with a white ethnic group were likely over-represented, highlighting the need to tackle ethnic inequality in digital health.^34,35^ Further, we did not evaluate all recently developed communication tools for multiple interventions and outcomes, which should be explored in future studies.^39,40^ We chose these interventions based on their widespread use (Cochrane’s summary of findings table), and the different approaches taken in using textual and graphical information. The analogies and differences across these interventions allowed us to infer about the effect of combining different sources of information on the communication of benefits and harms.

We acknowledge that the measured duration (between 15 and 20 minutes on average) is hardly compatible with the time constraints of routine clinical practice. However, the measured duration reflects provision of baseline data, familiarisation with the fictional scenario, and collection of outcome data. These additional steps would not occur in real-world practice, and we believe similar communication tools could be used in a more streamlined and targeted manner.

Finally, participants in the CICERO trial were asked to go through a scenario on fictional medications for a specific disorder (social anxiety) and to make a choice according to a given set of preferences. Such experimental framework may explain the overall performance in understanding medical information, although it is not possible to exclude that these figures are representative of existing challenges in understanding and trusting science. We believe that our findings are generalisable to medical conditions beyond mental health; however, the effect of these communication strategies when people are effectively making decisions for a condition that is relevant to them using their own set of values and preferences should be further explored. For instance, allowing a human-to-human interaction may allow further insights on the steps leading from information comprehension to being engaged with SDM.

## Conclusions

In an online RCT in the general population, we found that people allocated to the Kilim and Vitruvian plots experienced lower decisional conflict, better understood the provided evidence, and felt more confident about it and prepared to engage in decision-making compared to those allocated to the summary of findings table. The Vitruvian plot was associated with better understanding and lower preparedness to decision-making compared to the Kilim plot. We identified several important prognostic factors that demand tailored initiatives to address health inequalities.

## Supplementary Material

appendix

## Figures and Tables

**Table 1:** Characteristics of the enrolled participants. In the analyses, primary and secondary school were merged into a single category. Percentages do not always add up to 100 due to rounding. n = number; SD = standard deviation.

	Cochrane summary of findings (n = 507)	Kilim plot n = 603)	Vitruvian plot (n = 592)	Overall (n = 1,702)
**Age (mean ± SD)**	47.3 ± 16.1	48.3 ± 15.5	49.8 ± 14.6	48.5 ± 15.4
**Sex (n, %)**				
Female Male Prefer not to say	335 (70%) 149 (29%) 3 (1%)	404 (67%) 196 (33%) 3 (1%)	378 (64%) 213 (36%) 1 (0%)	1,137 (67%) 558 (33%) 7 (0%)
**Education (n, %)**				
Primary school Secondary school Upper school University Post-graduate Prefer not to say	0 (0%) 53 (11%) 124 (25%) 208 (41%) 118 (23%) 4 (1%)	0 (0%) 60 (10%) 136 (23%) 233 (39%) 171 (28%) 3 (1%)	2 (0%) 75 (13%) 132 (22%) 225 (38%) 156 (26%) 2 (0%)	2 (0%) 188 (11%) 392 (23%) 666 (39%) 445 (26%) 9 (1%)
**Ethnic group (n, %)**				
Arab Asian/Asian British Black Chinese Mixed/multiple White Other	1 (0%) 13 (3%) 4 (1%) 6 (1%) 11 (2%) 465 (92%) 7 (1%)	2 (0%) 26 (4%) 3 (1%) 4 (1%) 5 (1%) 557 (92%) 6 (1%)	2 (0%) 21 (4%) 6 (1%) 0 (0%) 13 (2%) 540 (91%) 10 (2%)	5 (0%) 60 (4%) 13 (1%) 10 (1%) 29 (2%) 1,562 (92%) 23 (1%)
**Literacy and numeracy (n, %)**				
Highly likely limited Possibly limited Adequate	21 (4%) 81 (16%) 405 (80%)	20 (3%) 86 (14%) 497 (82%)	17 (3%) 114 (19%) 461 (78%)	58 (3%) 281 (17%) 1,363 (80%)
**Lived experience of the medical condition of interest (social anxiety) (n, %)**				
No Yes Prefer not to say	404 (80%) 90 (18%) 13 (3%)	501 (83%) 92 (15%) 10 (2%)	463 (78%) 116 (20%) 13 (2%)	1,368 (80%) 298 (18%) 36 (2%)

**Table 2:** Performance of the three communication tools. Lower scores at DCS means less decisional conflict. Higher scores at DSE means higher self-efficacy. Higher scores at PDM means higher preparation for decision-making. aMD = adjusted mean difference; CI = confidence interval; DCS = Decisional Conflict Scale; DSE = Decision Self-Efficacy; IQR = InterQuartile Range; PDM = Preparation to Decision-Making; SD = Standard Deviation.

	Cochrane summary of findings table (n = 507)	Kilim plot (n = 603)	Vitruvian plot (n = 592)
***Primary outcome***			
**DCS (mean ± SD)** aMD (95%CI) aMD (95%CI)	32.1 ± 24.5 ref 9.7 (7.1 to 12.4)	21.9 ± 22.0 -9.7 (-12.4 to -7.1) ref	20.9 ± 21.6 -10.9 (-13.5 to -8.2) -1.1 (-3.7 to 1.4)
***Secondary outcomes***			
**DCS clarity (mean ± SD)**	20.0 ± 31.0	12.6 ± 26.6	13.0 ± 26.1
**DCS knowledge (mean ± SD)**	21.6 ± 27.7	13.7 ± 24.2	13.8 ± 24.6
**DCS support (mean ± SD)**	36.0 ± 28.9	25.7 ± 27.0	23.8 ± 26.0
**DCS uncertainty (mean ± SD)**	54.2 ± 37.9	37.9 ± 36.8	35.2 ± 34.9
**Information comprehension** Incorrect (n, %) Correct (n, %)	377 (74%) 130 (26%)	365 (61%) 238 (39%)	281 (47%) 311 (53%)
**DSE (mean ± SD)**	66.4 ± 25.7	75.1 ± 23.3	74.1 ± 23.7
**PDM (mean ± SD)**	59.2 ± 27.1	71.8 ± 23.9	68.3 ± 25.4
**Time, minutes (median, IQR)**	16.9 [12.3 to 24.1]	14.2 [10.3 to 18.9]	15.5 [11.3 to 21.7]

## Data Availability

Pseudonymised patient level data are available on reasonable request from the corresponding author after approval by the medical and health research ethics committee in the United Kingdom.

## Box 1 Cochrane summary of findings table

**Table:** Medication 1 versus placebo for social anxiety disorder (social phobia) Patient or population: people with social anxiety disorder (social phobia) Intervention: medication 1 Comparison: placebo

Outcomes	Illustrative comparative risks (95% CI)		Relative effect (95% CI)	Comments
	Assumed risk	Corresponding risk		
	Placebo	Medication 1		
**Response**	**16 per 100**	**24 per 100** (19 to 30)	**OR 1.69** (1.26 to 1.27)	
**Quality of life**	**16 per 100**	**23 per 100** (20 to 27)	**OR 1.60** (1.32 to 1.94)	
**Acceptability**	**11 per 100**	**11 per 100** (8 to 15)	**OR 0.97** (0.67 to 1.40)	
**Headache**	**2 per 100**	**3 per 100** (2 to 4)	**OR 1.10** (0.64 to 1.89)	
**Nausea**	**5 per 100**	**4 per 100** (2 to 10)	**OR 0.78** (0.30 to 2.07)	

**Table:** Medication 2 versus placebo for social anxiety disorder (social phobia) Patient or population: people with social anxiety disorder (social phobia) Intervention: medication 2 Comparison: placebo

Outcomes	Illustrative comparative risks (95% CI)		Relative effect (95% CI)	Comments
	Assumed risk	Corresponding risk		
	Placebo	Medication 2		
**Response**	**16 per 100**	**24 per 100** (19 to 30)	**OR 1.69** (1.26 to 1.27)	
**Quality of life**	**16 per 100**	**23 per 100** (20 to 27)	**OR 1.60** (1.32 to 1.94)	
**Acceptability**	**11 per 100**	**11 per 100** (10 to 11)	**OR 0.97** (0.93 to 1.01)	
**Headache**	**2 per 100**	**3 per 100** (2 to 3)	**OR 1.10** (1.02 to 1.20)	
**Nausea**	**5 per 100**	**4 per 100** (2 to 10)	**OR 0.78** (0.30 to 2.07)	

**Table:** Medication 3 versus placebo for social anxiety disorder (social phobia) Patient or population: people with social anxiety disorder (social phobia) Intervention: medication 3 Comparison: placebo

Outcomes	Illustrative comparative risks (95% CI)		Relative effect (95% CI)	Comments
	Assumed risk	Corresponding risk		
	Placebo	Medication 3		
**Response**	**16 per 100**	**24 per 100** (19 to 30)	**OR 1.69** (1.26 to 1.27)	
**Quality of life**	**16 per 100**	**23 per 100** (20 to 27)	**OR 1.60** (1.32 to 1.94)	
**Acceptability**	**11 per 100**	**9 per 100** (7 to 11)	**OR 0.81** (0.63 to 1.04)	
**Headache**	**2 per 100**	**3 per 100** (2 to 4)	**OR 1.10** (0.64 to 1.89)	
**Nausea**	**5 per 100**	**9 per 100** (5 to 16)	**OR 2.02** (1.11 to 3.67)	

**Table:** Medication 4 versus placebo for social anxiety disorder (social phobia) Patient or population: people with social anxiety disorder (social phobia) Intervention: medication 4 Comparison: placebo

Outcomes	Illustrative comparative risks (95% CI)		Relative effect (95% CI)	Comments
	Assumed risk	Corresponding risk		
	Placebo	Medication 4		
**Response**	**16 per 100**	**25 per 100** (20 to 30)	**OR 1.75** (1.33 to 2.32)	
**Quality of life**	See comment	See comment	Not estimable	No data available.
**Acceptability**	**11 per 100**	**11 per 100** (8 to 15)	**OR 0.97** (0.67 to 1.40)	
**Headache**	**2 per 100**	**3 per 100** (2 to 5)	**OR 1.29** (0.79 to 2.10)	
**Nausea**	**5 per 100**	**4 per 100** (2 to 10)	**OR 0.78** (0.30 to 2.07)	

**Table:** Medication 5 versus placebo for social anxiety disorder (social phobia) Patient or population: people with social anxiety disorder (social phobia) Intervention: medication 5 Comparison: placebo

Outcomes	Illustrative comparative risks (95% CI)		Relative effect (95% CI)	Comments
	Assumed risk	Corresponding risk		
	Placebo	Medication 5		
**Response**	**16 per 100**	**18 per 100** (17 to 20)	**OR 1.69** (1.26 to 1.27)	
**Quality of life**	**16 per 100**	**23 per 100** (20 to 27)	**OR 1.60** (1.32 to 1.94)	
**Acceptability**	**11 per 100**	**11 per 100** (10 to 11)	**OR 0.97** (0.94 to 1.01)	
**Headache**	**2 per 100**	**1 per 100** (1 to 2)	**OR 0.61** (0.55 to 0.68)	
**Nausea**	**5 per 100**	**4 per 100** (2 to 10)	**OR 0.78** (0.30 to 2.07)	

### Kilim plot

**Table:**

	Response	Quality of life	Acceptability	Headache	Nausea
Placebo	15.9%	15.9%	10.9%	2.3%	4.9%
Medication 1	24.0%	23.0%	11.0%	3.0%	4.3%
Medication 2	24.0%	23.0%	11.0%	3.1%	4.3%
Medication 3	24.0%	23.2%	9.2%	3.0%	9.1%
Medication 4	25.2%	-	11.3%	2.9%	3.9%
Medication 5	18.0%	23.0%	11.0%	1.3%	4.3%

### Vitruvian plot

***Please read the following scenario, and assume you are Mrs Jones***.

**Mrs Jones has social anxiety disorder (social phobia) and discussed it with her doctor. Mrs Jones is willing to start a medication that can help her feel better.**

- For Mrs Jones, the treatment must work well (*response*) and make her feel better (*quality of life*).
- Mrs Jones wants to be certain that she can continue the treatment. Therefore, she prefers certain over uncertain treatment effects when considering the likelihood of stopping the treatment (*acceptability*).
- Regarding side effects, Mrs Jones is mainly concerned about not experiencing nausea (*nausea*). She does not care about headaches (*headache*) and considers this irrelevant to make a choice.

**Set of multiple competing fictional medications across the three investigated communication tools.** Participants were provided with instructions for the tool they had been allocated to and asked to explore five possible competing interventions medications. We provided the comparator (placebo) for all the communication tools to contextualise absolute effects. For the Kilim and Vitruvian plots, vibrant green/red colours indicate strong statistical evidence of a beneficial/detrimental effect compared to the comparator. Light green/red colours indicate weaker evidence of a beneficial/detrimental effect. Colours closer to yellow indicate an increasing lack of evidence on whether the intervention performs better or worse than placebo. The light blue colour identifies placebo as the common comparator. CI = confidence interval; OR = odds ratio.
