# Supplementary material for: Communication of information on benefits and harms of multiple competing medical interventions: a three-group, open label randomised controlled trial
Source: Br J Psychiatry. Author manuscript; Available in PMC 2026 Sep 11. (PMC7619484; doi:10.1192/bjp.2026.10555)
Supplement: Supplementary file 1 [file EMS217907-supplement-appendix.docx]

**Communication of information on benefits and harms of multiple competing medical interventions: a three-group, open label randomised controlled trial**

Edoardo G Ostinelli, Orestis Efthimiou, Adeola Agunbiade, Huseyin Naci, Andrea Cipriani

Supplementary

INDEX

[1. SAMPLE SIZE REQUIREMENT	4](#_Toc183439000)

[2. STUDY DESIGN	5](#_Toc183439001)

[2.1 Study flow	5](#_Toc183439002)

[2.2 Definition of ethnic groups	6](#_Toc183439003)

[2.3 Process of communication of benefits and harms	7](#_Toc183439004)

[3. INSTRUCTIONS	8](#_Toc183439005)

[3.1 Summary of findings table	8](#_Toc183439006)

[3.2 Kilim plot	10](#_Toc183439007)

[3.3 Vitruvian plot	12](#_Toc183439008)

[4. INTERVENTIONS	16](#_Toc183439009)

[4.1 Summary of findings table	17](#_Toc183439010)

[4.2 Kilim plot	20](#_Toc183439011)

[4.3 Vitruvian plot	21](#_Toc183439012)

[5. SCENARIO	22](#_Toc183439013)

[6. RELATIVE EFFECTS BETWEEN INTERVENTIONS	23](#_Toc183439014)

[6.1 Decisional Conflict Scale, low literacy version (primary outcome)	23](#_Toc183439015)

[6.1.1 Summary (total score) – analysis	24](#_Toc183439016)

[6.1.2 Knowledge sub-scale – analysis	26](#_Toc183439017)

[6.1.3 Certainty sub-scale – analysis	28](#_Toc183439018)

[6.1.4 Values clarity sub-scale – analysis	30](#_Toc183439019)

[6.1.5 Support sub-scale – analysis	32](#_Toc183439020)

[6.2 Information comprehension (secondary outcome)	34](#_Toc183439021)

[6.2.1 Summary – analysis	37](#_Toc183439022)

[6.3 Decision Self-Efficacy scale (secondary outcome)	39](#_Toc183439023)

[6.3.1 Summary (total score) – analysis	40](#_Toc183439024)

[6.4 Preparation for Decision Making scale (secondary outcome)	42](#_Toc183439025)

[6.4.1 Summary (total score) – analysis	43](#_Toc183439026)

[6.5 Time to completion (secondary outcome)	45](#_Toc183439027)

[6.5.1 Time to completion (secondary outcome) – without outliers	46](#_Toc183439028)

[6.5.2 Time to completion (secondary outcome) – with outliers	47](#_Toc183439029)

[6.6 Choice with participants’ own set of preferences (additional analysis)	48](#_Toc183439030)

[6.7 Drop-out (additional analysis)	49](#_Toc183439031)

[6.8 Overview of prognostic factors	50](#_Toc183439032)

[7. ASSOCIATIONS/CAUSAL PATHWAYS BETWEEN OUTCOMES	52](#_Toc183439033)

[7.1 Outcomes correlation	52](#_Toc183439034)

[7.1.1 Outcomes correlation (with outliers)	53](#_Toc183439035)

[7.1.2 Outcomes correlation (without outliers)	55](#_Toc183439036)

[7.2 Information comprehension (association with other outcomes)	57](#_Toc183439037)

[7.2.1 Decisional Conflict Scale low literacy (total score)	57](#_Toc183439038)

[7.2.2 Decision Self-Efficacy (total score)	58](#_Toc183439039)

[7.2.3 Preparation for Decision-Making (total score)	59](#_Toc183439040)

[7.2.4 Time to completion (minutes)	60](#_Toc183439041)

[7.3 Information comprehension	61](#_Toc183439042)

# 1. SAMPLE SIZE REQUIREMENT

Based on previous trials evaluating patient decision aid tools to communicate benefit and harm information (Hopkin et al. 2019), we considered a mean endpoint score of 23.13 and 14.59 and a standard deviation of 20.34 and 15.04 at the Decision Conflict Scale low literacy version (primary outcome) for experimental and control arms. Assuming 3 groups, a 90% power and a significance level set as $0.05/3$ (Bonferroni correction for multiple comparisons), each arm will require 120 patients (Hopkin et al. 2019; Pocock 1983). Sample size calculations were done in R using the *pwr* package. Borrowing a drop-out rate of 0.22 (participants randomised that dropped out), the total sample size of the trial is 462 participants (154 participants per arm).

# 2. STUDY DESIGN

## 2.1 Study flow

Reported as submitted in the documents to the Ethical committee (section E2):

1. Potential participants read additional information on the CICERO trial on a dedicated webpage hosted on the Departmental website;
2. If interested in the CICERO trial, Potential participants can access an online version of the PIS;
3. Potential participants are asked to reflect on the PIS for enough time (without any time limitation), without any need to feel obliged to continue;
4. If and when potential participants feel ready to take part in the CICERO trial, they will click on a link that will redirect them to the website to collect their consent.
5. Potential participants will express their consent to take part in the CICERO trial and confirm that they meet the eligibility criteria.
6. Participants will be randomised to one of three interventions (Vitruvian plot; Kilim plot; Summary of Findings table) and redirected to the relevant online form on JISC. The three forms will include an image of the intervention (based on the allocation) and the questionnaires (identical in all three forms).
7. After the online forms have been completed (expected time to completion: around 20 minutes), the study session and their journey in the CICERO trial is over.

## 2.2 Definition of ethnic groups

We collected data on self-identified ethnic groups or backgrounds following the harmonised macro-categories country-specific ethnic group questions for England and Wales as defined by the United Kingdom Office for National Statistics (<https://www.ons.gov.uk/methodology/classificationsandstandards/measuringequality/ethnicgroupnationalidentityandreligion#ethnic-group>, “Ethnic group question for telephone interview (as applied on the LFS)”, part 1, ETH11, “In England and Wales”):

- White
- Mixed/Multiple ethnic groups
- Asian/Asian British
- Black/African/Caribbean/Black British
- Chinese
- Arab
- Other ethnic group

Participants selecting “Other ethnic group” could specify their ethnic group or background in a free-text field.

## 2.3 Process of communication of benefits and harms

The process about communicating information on benefits and harms involves three key steps: firstly, understanding the available information; secondly, feeling confident about one’s own understanding; and finally, feeling prepared to actively participate in shared decision-making.

We used the Decisional Conflict Scale (low literacy version) to measure the overall subjective effectiveness of the communication process, as well as each step individually. Information comprehension, Decision Self-Efficacy scale and the Preparation for Decision Making scale are positive measures (i.e. the higher the number, the better it is), while Decisional Conflict Scale is a negative measure (i.e. the higher the number, the worse it is).

# 3. INSTRUCTIONS

## 3.1 Summary of findings table

On the next page, you will be presented with a clinical scenario and information about available treatments. You will also see information about the comparator group (placebo).

⚠️ Read carefully the following instruction to familiarise yourself with the table (communication tool). You will be able to return to this section at any time. You can also download this explanation by clicking [here].

How to read the communication tool

Each table provides information on one medication. For example, the table below is about "medication 4".

| **Medication 4 versus placebo for social anxiety disorder (social phobia)**  Patient or population: people with social anxiety disorder (social phobia)  Intervention: medication 4  Comparison: placebo | | | | |
| --- | --- | --- | --- | --- |
| Outcomes | **Illustrative comparative risks** (95% CI) | | Relative effect (95% CI) | Comments |
|  | Assumed risk | Corresponding risk |  |  |
|  | **Placebo** | **Medication 4** |  |  |
| **Response** | **16 per 100** | **25 per 100** (20 to 30) | **OR 1.75** (1.33 to 2.32) |  |
| **Quality of life** | See comment | See comment | Not estimable | No data available. |
| **Acceptability** | **11 per 100** | **11 per 100** (8 to 15) | **OR 0.97** (0.67 to 1.40) |  |
| **Headache** | **2 per 100** | **3 per 100** (2 to 5) | **OR 1.29** (0.79 to 2.10) |  |
| **Nausea** | **5 per 100** | **4 per 100** (2 to 10) | **OR 0.78** (0.30 to 2.07) |  |

Each row represents one of five outcomes: "response", "quality of life", "acceptability", "headache", and "nausea".

**If no information is available for one outcome, the cells will be empty**. No information is available for "quality of life" in the table above.

The assumed risk with placebo tells you what happens to people taking a pill without pharmacological effect.

**The higher the corresponding risk for medication 4, the greater the effect**. For instance, on average, 25% people that take "medication 4" experience a "response" (25 per 100).

**The numbers below the corresponding risk represent how certain the effect is compared to the assumed risk with placebo**. For instance, we are quite certain (sure) about how well "medication 4" works in achieving "response" (20 to 30).

On the other hand, we are uncertain (not sure) about the bad effect on "headache" for "medication 4" (2 to 5).

## 3.2 Kilim plot

On the next page, you will be presented with a clinical scenario and information about available treatments. You will also see information about the comparator group (placebo).

⚠️ Read carefully the following instruction to familiarise yourself with the table (communication tool). You will be able to return to this section at any time. You can also download this explanation by clicking [here].

How to read the communication tool

Each row provides information on one medication. For example, the table below is about "medication 4".

|  | Response | Quality of life | Acceptability | Headache | Nausea |
| --- | --- | --- | --- | --- | --- |
| Medication 4 | 25.2% | - | 11.3% | 2.9% | 3.9% |

Each column represents one of five outcomes: "response", "quality of life", "acceptability", "headache", and "nausea".

**If no information is available for one outcome, the cell will be empty and the colour will change to white**. No information is available for "quality of life" in the row above.

**The higher the number, the greater the effect**. For instance, on average, 25.2% of the people that take "medication 4" (25 out of 100 people) experience a "response”.

**Green is good, red is bad** when comparing "medication 4" to placebo. For instance, "medication 4" seems to be associated with less "nausea" than placebo (green).

**The more vibrant the green or red cells are, the more certain the effect is**. For instance, we are quite certain (sure) about how well "medication 4" works in achieving "response" (vibrant green).

**When information on an outcome is uncertain, the colour will lighten** (pale/light green or pale/light red). Maximum uncertainty is represented by pale/light yellow. For instance, we are uncertain (not sure) about the bad effect on "headache" for "medication 4" (light red instead of vibrant red).

Comparator (placebo)

The placebo row tells you what happens to people taking a pill without pharmacological effect.

|  | Response | Quality of life | Acceptability | Headache | Nausea |
| --- | --- | --- | --- | --- | --- |
| Placebo | 15.9% | 15.9% | 10.9% | 2.3% | 4.9% |

**All the cells (outcomes) are light blue** since placebo is for comparison only.

## 3.3 Vitruvian plot

On the next page, you will be presented with a clinical scenario and information about available treatments. You will also see information about the comparator group (placebo).

⚠️ Read carefully the following instruction to familiarise yourself with the plot (communication tool). You will be able to return to this section at any time. You can also download this explanation by clicking [here].

How to read the communication tool

Each plot provides information on one medication. For example, the plot below is about "medication 4".

Each section represents one of five outcomes: "response", "quality of life", "acceptability", "headache", and "nausea".

**If no information is available for one outcome, the section will be empty, and the outer ring's border will change to white** (instead of light blue). No information is available for "quality of life" in the plot above.

**The bigger the coloured slice, the greater the effect** (also reported as a number in the grey box). For instance, on average, 25% of the people that take "medication 4" (25 out of 100 people) experience a "response" (big slice).

**Green is good, red is bad** when comparing "medication 4" to placebo. For instance, "medication 4" seems to be associated with less "nausea" than placebo (green).

**The more vibrant the green or red slices are, the more certain the effect is**. For instance, we are quite certain (sure) about how well "medication 4" works in achieving "response" (vibrant green).

**When information on an outcome is uncertain, the colour will lighten** (pale/light green or pale/light red). Maximum uncertainty is represented by pale/light yellow. For instance, we are uncertain (not sure) about the bad effect on "headache" for "medication 4" (light red instead of vibrant red).

**Comparator (placebo)**

The placebo plot tells you what happens to people taking a pill without pharmacological effect.

**All the slices (outcomes) are light blue** since placebo is for comparison only.

# 4. INTERVENTIONS

**Characteristics of the three communication tools**

The Cochrane summary of findings table, Kilim plot, and Vitruvian plot differ in how several domains of benefits and harms information are communicated.

|  | **Cochrane summary of findings table** | **Kilim plot** | **Vitruvian plot** |
| --- | --- | --- | --- |
| Magnitude of effect |   textual |   textual |    graphical and textual |
| Direction of effect |   textual |    graphical and textual |    graphical and textual |
| Certainty of effect |   textual |   graphical |   graphical |
| Availability of evidence |   textual |   textual |    graphical and textual |

## 4.1 Summary of findings table

| **Medication 1 versus placebo for social anxiety disorder (social phobia)**  Patient or population: people with social anxiety disorder (social phobia)  Intervention: medication 1  Comparison: placebo | | | | |
| --- | --- | --- | --- | --- |
| Outcomes | **Illustrative comparative risks** (95% CI) | | Relative effect (95% CI) | Comments |
|  | Assumed risk | Corresponding risk |  |  |
|  | **Placebo** | **Medication 1** |  |  |
| **Response** | **16 per 100** | **24 per 100** (19 to 30) | **OR 1.69** (1.26 to 1.27) |  |
| **Quality of life** | **16 per 100** | **23 per 100** (20 to 27) | **OR 1.60** (1.32 to 1.94) |  |
| **Acceptability** | **11 per 100** | **11 per 100** (8 to 15) | **OR 0.97** (0.67 to 1.40) |  |
| **Headache** | **2 per 100** | **3 per 100** (2 to 4) | **OR 1.10** (0.64 to 1.89) |  |
| **Nausea** | **5 per 100** | **4 per 100** (2 to 10) | **OR 0.78** (0.30 to 2.07) |  |

| **Medication 2 versus placebo for social anxiety disorder (social phobia)**  Patient or population: people with social anxiety disorder (social phobia)  Intervention: medication 2  Comparison: placebo | | | | |
| --- | --- | --- | --- | --- |
| Outcomes | **Illustrative comparative risks** (95% CI) | | Relative effect (95% CI) | Comments |
|  | Assumed risk | Corresponding risk |  |  |
|  | **Placebo** | **Medication 2** |  |  |
| **Response** | **16 per 100** | **24 per 100** (19 to 30) | **OR 1.69** (1.26 to 1.27) |  |
| **Quality of life** | **16 per 100** | **23 per 100** (20 to 27) | **OR 1.60** (1.32 to 1.94) |  |
| **Acceptability** | **11 per 100** | **11 per 100** (10 to 11) | **OR 0.97** (0.93 to 1.01) |  |
| **Headache** | **2 per 100** | **3 per 100** (2 to 3) | **OR 1.10** (1.02 to 1.20) |  |
| **Nausea** | **5 per 100** | **4 per 100** (2 to 10) | **OR 0.78** (0.30 to 2.07) |  |

| **Medication 3 versus placebo for social anxiety disorder (social phobia)**  Patient or population: people with social anxiety disorder (social phobia)  Intervention: medication 3  Comparison: placebo | | | | |
| --- | --- | --- | --- | --- |
| Outcomes | **Illustrative comparative risks** (95% CI) | | Relative effect (95% CI) | Comments |
|  | Assumed risk | Corresponding risk |  |  |
|  | **Placebo** | **Medication 3** |  |  |
| **Response** | **16 per 100** | **24 per 100** (19 to 30) | **OR 1.69** (1.26 to 1.27) |  |
| **Quality of life** | **16 per 100** | **23 per 100** (20 to 27) | **OR 1.60** (1.32 to 1.94) |  |
| **Acceptability** | **11 per 100** | **9 per 100** (7 to 11) | **OR 0.81** (0.63 to 1.04) |  |
| **Headache** | **2 per 100** | **3 per 100** (2 to 4) | **OR 1.10** (0.64 to 1.89) |  |
| **Nausea** | **5 per 100** | **9 per 100** (5 to 16) | **OR 2.02** (1.11 to 3.67) |  |

| **Medication 4 versus placebo for social anxiety disorder (social phobia)**  Patient or population: people with social anxiety disorder (social phobia)  Intervention: medication 4  Comparison: placebo | | | | |
| --- | --- | --- | --- | --- |
| Outcomes | **Illustrative comparative risks** (95% CI) | | Relative effect (95% CI) | Comments |
|  | Assumed risk | Corresponding risk |  |  |
|  | **Placebo** | **Medication 4** |  |  |
| **Response** | **16 per 100** | **25 per 100** (20 to 30) | **OR 1.75** (1.33 to 2.32) |  |
| **Quality of life** | See comment | See comment | Not estimable | No data available. |
| **Acceptability** | **11 per 100** | **11 per 100** (8 to 15) | **OR 0.97** (0.67 to 1.40) |  |
| **Headache** | **2 per 100** | **3 per 100** (2 to 5) | **OR 1.29** (0.79 to 2.10) |  |
| **Nausea** | **5 per 100** | **4 per 100** (2 to 10) | **OR 0.78** (0.30 to 2.07) |  |

| **Medication 5 versus placebo for social anxiety disorder (social phobia)**  Patient or population: people with social anxiety disorder (social phobia)  Intervention: medication 5  Comparison: placebo | | | | |
| --- | --- | --- | --- | --- |
| Outcomes | **Illustrative comparative risks** (95% CI) | | Relative effect (95% CI) | Comments |
|  | Assumed risk | Corresponding risk |  |  |
|  | **Placebo** | **Medication 5** |  |  |
| **Response** | **16 per 100** | **18 per 100** (17 to 20) | **OR 1.69** (1.26 to 1.27) |  |
| **Quality of life** | **16 per 100** | **23 per 100** (20 to 27) | **OR 1.60** (1.32 to 1.94) |  |
| **Acceptability** | **11 per 100** | **11 per 100** (10 to 11) | **OR 0.97** (0.94 to 1.01) |  |
| **Headache** | **2 per 100** | **1 per 100** (1 to 2) | **OR 0.61** (0.55 to 0.68) |  |
| **Nausea** | **5 per 100** | **4 per 100** (2 to 10) | **OR 0.78** (0.30 to 2.07) |  |

## 4.2 Kilim plot

|  | Response | Quality of life | Acceptability | Headache | Nausea |
| --- | --- | --- | --- | --- | --- |
| Placebo | 15.9% | 15.9% | 10.9% | 2.3% | 4.9% |
| Medication 1 | 24.0% | 23.0% | 11.0% | 3.0% | 4.3% |
| Medication 2 | 24.0% | 23.0% | 11.0% | 3.1% | 4.3% |
| Medication 3 | 24.0% | 23.2% | 9.2% | 3.0% | 9.1% |
| Medication 4 | 25.2% | - | 11.3% | 2.9% | 3.9% |
| Medication 5 | 18.0% | 23.0% | 11.0% | 1.3% | 4.3% |

## 4.3 Vitruvian plot

# 5. SCENARIO

***Please read the following scenario, and assume you are Mrs Jones.***

Mrs Jones has social anxiety disorder (social phobia) and discussed it with her doctor. Mrs Jones is willing to start a medication that can help her feel better.

- For Mrs Jones, the treatment must work well (*response*) and make her feel better (*quality of life*).
- Mrs Jones wants to be certain that she can continue the treatment. Therefore, she prefers certain over uncertain treatment effects when considering the likelihood of stopping the treatment (*acceptability*).
- Regarding side effects, Mrs Jones is mainly concerned about not experiencing nausea (*nausea*). She does not care about headaches (*headache*) and considers this irrelevant to make a choice.

⬇️ Compare the following medications (if you want, you can return to the previous page and read the instructions again).

⚠️ If the image quality is insufficient, please open this link in another tab: [[URL]](https://cicerotrial.s3.eu-west-2.amazonaws.com/synoptic+chart.png).

[communication tool]

**Based on her preferences, which medication should Mrs Jones take? (assume she can access any of the medications for free.)**

- Medication 1
- Medication 2
- Medication 3
- Medication 4
- Medication 5
- I don’t know

Adapted from Schwartz LM, Woloshin S, Welch HG. The drug facts box: providing consumers with simple tabular data on drug benefit and harm. Med Decis Making. 2007 Sep-Oct;27(5):655-62. doi: 10.1177/0272989X07306786. Epub 2007 Sep 14. PMID: 17873258.

# 6. RELATIVE EFFECTS BETWEEN INTERVENTIONS

## 6.1 Decisional Conflict Scale, low literacy version (primary outcome)

The Decisional Conflict Scale (DCS) measures perceptions of difficulty in making decisions, including lack of information to help choose between options and feeling unclear about one’s own preferences (O’Connor 1993). It is a commonly used rating scale to evaluate the overall performance of PDAs and communication of benefit-harm information (Hopkin et al. 2019, Stacey et al. 2017). The scale has 10 items, rated along a 3-point scale with options of ‘‘yes,’’ ‘‘unsure,’’ and ‘‘no,’’ and the combined scores are converted to a 0 to 100 scale, with lower scores indicating lower levels of decision conflict. The scale has been validated previously and had an acceptable alpha score of 0.76 (Linder et al. 2011).

**References**

- Hopkin G, Au A, Collier VJ, Yudkin JS, Basu S, Naci H. Combining Multiple Treatment Comparisons with Personalized Patient Preferences: A Randomized Trial of an Interactive Platform for Statin Treatment Selection. Med Decis Making. 2019 Apr;39(3):264-277. doi: 10.1177/0272989X19835239. Epub 2019 Mar 15. PMID: 30873906.
- Linder SK, Swank PR, Vernon SW, Mullen PD, Morgan RO, Volk RJ. Validity of a low literacy version of the Decisional Conflict Scale. Patient Educ Couns. 2011 Dec;85(3):521-4. doi: 10.1016/j.pec.2010.12.012. Epub 2011 Feb 5. PMID: 21300518; PMCID: PMC3121898.
- O’Connor AM. User Manual - Decisional Conflict Scale (10 item question format) [document on the Internet]. Ottawa: Ottawa Hospital Research Institute; 1993 [updated 2010].
- Stacey D, Lewis KB, Smith M, Carley M, Volk R, Douglas EE, Pacheco-Brousseau L, Finderup J, Gunderson J, Barry MJ, Bennett CL, Bravo P, Steffensen K, Gogovor A, Graham ID, Kelly SE, Légaré F, Sondergaard H, Thomson R, Trenaman L, Trevena L. Decision aids for people facing health treatment or screening decisions. Cochrane Database Syst Rev. 2024 Jan 29;1(1):CD001431. doi: 10.1002/14651858.CD001431.pub6. PMID: 38284415; PMCID: PMC10823577.

### 6.1.1 Summary (total score) – analysis

| Variable | Estimate | Std. Error | t value | Pr(>\|t\|) |
| --- | --- | --- | --- | --- |
| (Intercept) | 46.30969 | 3.52597 | 13.134 | < 2e-16 *** |
| Intervention - Kilim | -9.72366 | 1.3413 | -7.249 | 6.35e-13 *** |
| Intervention – Vitruvian plot | -10.85852 | 1.35069 | -8.039 | 1.69e-15 *** |
| Sex - I do not know / prefer not to say | 10.98436 | 8.51117 | 1.291 | 0.19703 |
| Sex – male | 0.60155 | 1.16587 | 0.516 | 0.60594 |
| Ethnicity - Arab | -13.83597 | 10.00463 | -1.383 | 0.16686 |
| Ethnicity - Asian/Asian British | 3.06792 | 2.98241 | 1.029 | 0.30378 |
| Ethnicity - Black/African/Caribbean/Black British | 15.81188 | 6.18273 | 2.557 | 0.01063 * |
| Ethnicity - Chinese | -11.36292 | 7.05477 | -1.611 | 0.10744 |
| Ethnicity - Mixed/Multiple ethnic groups | 7.00776 | 4.17985 | 1.677 | 0.09381 . |
| Ethnicity - Other | 1.71915 | 4.67979 | 0.367 | 0.71340 |
| Age | -0.23515 | 0.03889 | -6.047 | 1.81e-09 *** |
| NVS – total score | -1.43701 | 0.48232 | -2.979 | 0.00293 ** |
| Education - Higher or secondary or further education (A-levels, BTEC, etc.) | 1.72367 | 1.99363 | 0.865 | 0.38739 |
| Education - College or university or equivalent | 1.20179 | 1.83316 | 0.656 | 0.51218 |
| Education - Post-graduate degree or equivalent | 3.42177 | 1.93793 | 1.766 | 0.07763 . |
| Education - I do not know / prefer not to say | 3.29652 | 7.65654 | 0.431 | 0.66685 |
| Familiarity - I do not know / prefer not to say | 6.99868 | 3.7833 | 1.85 | 0.06451 . |
| Familiarity - Yes | 2.98408 | 1.50922 | 1.977 | 0.04818 * |

Signif. codes: 0 ‘***’ 0.001 ‘**’ 0.01 ‘*’ 0.05 ‘.’ 0.1 ‘ ’ 1

Residual standard error: 22.15 on 1683 degrees of freedom

Multiple R-squared: 0.09366,	Adjusted R-squared: 0.08397

F-statistic: 9.662 on 18 and 1683 DF, p-value: < 2.2e-16

| Variable | **2.5%** | **97.5%** |
| --- | --- | --- |
| (Intercept) | 39.39393235 | 53.2254430 |
| Intervention - Kilim | -12.35445917 | -7.0928584 |
| Intervention – Vitruvian plot | -13.50773150 | -8.2093091 |
| Sex - I do not know / prefer not to say | -5.70923031 | 27.6779575 |
| Sex – male | -1.68515165 | 2.8882506 |
| Ethnicity - Arab | -33.45879117 | 5.7868461 |
| Ethnicity - Asian/Asian British | -2.78170366 | 8.9175398 |
| Ethnicity - Black/African/Caribbean/Black British | 3.68523480 | 27.9385210 |
| Ethnicity - Chinese | -25.19995908 | 2.4741245 |
| Ethnicity - Mixed/Multiple ethnic groups | -1.19049198 | 15.2060059 |
| Ethnicity - Other | -7.45967296 | 10.8979779 |
| Age | -0.31142475 | -0.1588783 |
| NVS – total score | -2.38303004 | -0.4909953 |
| Education - Higher or secondary or further education (A-levels, BTEC, etc.) | -2.18659080 | 5.6339283 |
| Education - College or university or equivalent | -2.39372325 | 4.7972955 |
| Education - Post-graduate degree or equivalent | -0.37922714 | 7.2227673 |
| Education - I do not know / prefer not to say | -11.72082753 | 18.3138672 |
| Familiarity - I do not know / prefer not to say | -0.42179026 | 14.4191555 |
| Familiarity - Yes | 0.02393957 | 5.9442153 |

### 6.1.2 Knowledge sub-scale – analysis

| Variable | Estimate | Std. Error | t value | Pr(>\|t\|) |
| --- | --- | --- | --- | --- |
| (Intercept) | 41.70484 | 3.93796 | 10.59 | < 2e-16 *** |
| Intervention - Kilim | -7.00731 | 1.49803 | -4.678 | 3.13e-06 *** |
| Intervention – Vitruvian plot | -7.41816 | 1.50851 | -4.918 | 9.62e-07 *** |
| Sex - I do not know / prefer not to say | 1.74192 | 9.50565 | 0.183 | 0.85462 |
| Sex – male | -0.2656 | 1.30209 | -0.204 | 0.83840 |
| Ethnicity - Arab | -13.64245 | 11.17361 | -1.221 | 0.22228 |
| Ethnicity - Asian/Asian British | 4.16016 | 3.33089 | 1.249 | 0.21185 |
| Ethnicity - Black/African/Caribbean/Black British | 22.58606 | 6.90514 | 3.271 | 0.00109 ** |
| Ethnicity - Chinese | -4.47882 | 7.87908 | -0.568 | 0.56981 |
| Ethnicity - Mixed/Multiple ethnic groups | 12.29088 | 4.66824 | 2.633 | 0.00854 ** |
| Ethnicity - Other | 6.17256 | 5.2266 | 1.181 | 0.23777 |
| Age | -0.22042 | 0.04343 | -5.075 | 4.30e-07 *** |
| NVS – total score | -2.32437 | 0.53868 | -4.315 | 1.69e-05 *** |
| Education - Higher or secondary or further education (A-levels, BTEC, etc.) | -1.05386 | 2.22658 | -0.473 | 0.63605 |
| Education - College or university or equivalent | -2.22985 | 2.04735 | -1.089 | 0.27625 |
| Education - Post-graduate degree or equivalent | -2.11927 | 2.16436 | -0.979 | 0.32764 |
| Education - I do not know / prefer not to say | 4.12086 | 8.55117 | 0.482 | 0.62994 |
| Familiarity - I do not know / prefer not to say | 7.31445 | 4.22536 | 1.731 | 0.08362 . |
| Familiarity - Yes | 5.2292 | 1.68556 | 3.102 | 0.00195 ** |

Signif. codes: 0 ‘***’ 0.001 ‘**’ 0.01 ‘*’ 0.05 ‘.’ 0.1 ‘ ’ 1

Residual standard error: 24.74 on 1683 degrees of freedom

Multiple R-squared: 0.07965,	Adjusted R-squared: 0.0698

F-statistic: 8.092 on 18 and 1683 DF, p-value: < 2.2e-16

| Variable | 2.5 % | 97.5 % |
| --- | --- | --- |
| (Intercept) | 33.9810202 | 49.4286672 |
| Intervention - Kilim | -9.9455084 | -4.0691184 |
| Intervention – Vitruvian plot | -10.3769121 | -4.459398 |
| Sex - I do not know / prefer not to say | -16.9022279 | 20.3860703 |
| Sex – male | -2.8194878 | 2.2882915 |
| Ethnicity - Arab | -35.5580851 | 8.2731902 |
| Ethnicity - Asian/Asian British | -2.3729539 | 10.6932822 |
| Ethnicity - Black/African/Caribbean/Black British | 9.0424816 | 36.1296316 |
| Ethnicity - Chinese | -19.9326488 | 10.975 |
| Ethnicity - Mixed/Multiple ethnic groups | 3.1347149 | 21.4470539 |
| Ethnicity - Other | -4.0787611 | 16.4238809 |
| Age | -0.3056024 | -0.1352318 |
| NVS – total score | -3.3809216 | -1.267813 |
| Education - Higher or secondary or further education (A-levels, BTEC, etc.) | -5.4210144 | 3.3132896 |
| Education - College or university or equivalent | -6.245477 | 1.7857729 |
| Education - Post-graduate degree or equivalent | -6.3643917 | 2.1258542 |
| Education - I do not know / prefer not to say | -12.6511789 | 20.8929056 |
| Familiarity - I do not know / prefer not to say | -0.973069 | 15.6019602 |
| Familiarity - Yes | 1.9231869 | 8.5352145 |

### 6.1.3 Certainty sub-scale – analysis

| Variable | Estimate | Std. Error | t value | Pr(>\|t\|) |
| --- | --- | --- | --- | --- |
| (Intercept) | 70.17348 | 5.7148 | 12.279 | < 2e-16 *** |
| Intervention - Kilim | -16.17905 | 2.17395 | -7.442 | 1.57e-13 *** |
| Intervention – Vitruvian plot | -18.41521 | 2.18916 | -8.412 | < 2e-16 *** |
| Sex - I do not know / prefer not to say | 11.66534 | 13.79466 | 0.846 | 0.39787 |
| Sex – male | 0.34837 | 1.8896 | 0.184 | 0.85375 |
| Ethnicity - Arab | -9.38583 | 16.21521 | -0.579 | 0.56278 |
| Ethnicity - Asian/Asian British | 6.08142 | 4.8338 | 1.258 | 0.20853 |
| Ethnicity - Black/African/Caribbean/Black British | -0.0915 | 10.02079 | -0.009 | 0.99272 |
| Ethnicity - Chinese | -32.87624 | 11.43416 | -2.875 | 0.00409 ** |
| Ethnicity - Mixed/Multiple ethnic groups | -3.67954 | 6.77458 | -0.543 | 0.58711 |
| Ethnicity - Other | -7.17554 | 7.58487 | -0.946 | 0.34427 |
| Age | -0.39116 | 0.06303 | -6.206 | 6.82e-10 *** |
| NVS – total score | -0.4468 | 0.78174 | -0.572 | 0.56771 |
| Education - Higher or secondary or further education (A-levels, BTEC, etc.) | 3.96815 | 3.23122 | 1.228 | 0.21959 |
| Education - College or university or equivalent | 3.67155 | 2.97113 | 1.236 | 0.21673 |
| Education - Post-graduate degree or equivalent | 6.05806 | 3.14093 | 1.929 | 0.05393 . |
| Education - I do not know / prefer not to say | 8.21403 | 12.4095 | 0.662 | 0.50812 |
| Familiarity - I do not know / prefer not to say | 8.45118 | 6.13187 | 1.378 | 0.16831 |
| Familiarity - Yes | 2.7376 | 2.44609 | 1.119 | 0.26323 |

```
---
Signif. codes:  0 ‘***’ 0.001 ‘**’ 0.01 ‘*’ 0.05 ‘.’ 0.1 ‘ ’ 1

Residual standard error: 35.9 on 1683 degrees of freedom
Multiple R-squared:  0.08645,	Adjusted R-squared:  0.07668
F-statistic: 8.848 on 18 and 1683 DF,  p-value: < 2.2e-16
```

| Variable | 2.5 % | 97.5 % |
| --- | --- | --- |
| (Intercept) | 58.9646216 | 81.3823367 |
| Intervention - Kilim | -20.4429714 | -11.91512 |
| Intervention – Vitruvian plot | -22.7089706 | -14.1214397 |
| Sex - I do not know / prefer not to say | -15.3911606 | 38.7218346 |
| Sex – male | -3.3578477 | 4.0545913 |
| Ethnicity - Arab | -41.1899278 | 22.4182739 |
| Ethnicity - Asian/Asian British | -3.3994781 | 15.5623197 |
| Ethnicity - Black/African/Caribbean/Black British | -19.7460126 | 19.563017 |
| Ethnicity - Chinese | -55.3029208 | -10.4495611 |
| Ethnicity - Mixed/Multiple ethnic groups | -16.9670309 | 9.6079419 |
| Ethnicity - Other | -22.0523185 | 7.7012348 |
| Age | -0.5147846 | -0.2675417 |
| NVS – total score | -1.9800745 | 1.086481 |
| Education - Higher or secondary or further education (A-levels, BTEC, etc.) | -2.3694848 | 10.3057879 |
| Education - College or university or equivalent | -2.1559459 | 9.4990508 |
| Education - Post-graduate degree or equivalent | -0.1024892 | 12.218605 |
| Education - I do not know / prefer not to say | -16.1256578 | 32.5537128 |
| Familiarity - I do not know / prefer not to say | -3.5757122 | 20.4780665 |
| Familiarity - Yes | -2.060111 | 7.5353019 |

### 6.1.4 Values clarity sub-scale – analysis

| Variable | Estimate | Std. Error | t value | Pr(>\|t\|) |
| --- | --- | --- | --- | --- |
| (Intercept) | 37.68903 | 4.36422 | 8.636 | < 2e-16 *** |
| Intervention - Kilim | -6.90731 | 1.66018 | -4.161 | 3.33e-05 *** |
| Intervention – Vitruvian plot | -6.74837 | 1.6718 | -4.037 | 5.67e-05 *** |
| Sex - I do not know / prefer not to say | 17.48697 | 10.53457 | 1.66 | 0.09711 . |
| Sex – male | 2.85598 | 1.44303 | 1.979 | 0.04796 * |
| Ethnicity - Arab | -17.17156 | 12.38307 | -1.387 | 0.16572 |
| Ethnicity - Asian/Asian British | 0.80316 | 3.69143 | 0.218 | 0.82779 |
| Ethnicity - Black/African/Caribbean/Black British | 21.58529 | 7.65257 | 2.821 | 0.00485 ** |
| Ethnicity - Chinese | -7.89036 | 8.73193 | -0.904 | 0.36633 |
| Ethnicity - Mixed/Multiple ethnic groups | 9.41355 | 5.17354 | 1.82 | 0.06900 . |
| Ethnicity - Other | 3.7272 | 5.79234 | 0.643 | 0.52001 |
| Age | -0.24578 | 0.04813 | -5.106 | 3.66e-07 *** |
| NVS – total score | -1.83481 | 0.59699 | -3.073 | 0.00215 ** |
| Education - Higher or secondary or further education (A-levels, BTEC, etc.) | 0.18163 | 2.46759 | 0.074 | 0.94133 |
| Education - College or university or equivalent | -0.49281 | 2.26896 | -0.217 | 0.82808 |
| Education - Post-graduate degree or equivalent | 1.86358 | 2.39864 | 0.777 | 0.43731 |
| Education - I do not know / prefer not to say | -7.95448 | 9.47677 | -0.839 | 0.40138 |
| Familiarity - I do not know / prefer not to say | 5.60176 | 4.68272 | 1.196 | 0.23176 |
| Familiarity - Yes | -0.35993 | 1.86801 | -0.193 | 0.84723 |

```
Signif. codes:  0 ‘***’ 0.001 ‘**’ 0.01 ‘*’ 0.05 ‘.’ 0.1 ‘ ’ 1

Residual standard error: 27.42 on 1683 degrees of freedom
Multiple R-squared:  0.04863,	Adjusted R-squared:  0.03846
F-statistic:  4.78 on 18 and 1683 DF,  p-value: 1.497e-10
```

| Variable | 2.5 % | 97.5 % |
| --- | --- | --- |
| (Intercept) | 29.1291623 | 46.2488973 |
| Intervention - Kilim | -10.16354025 | -3.6510766 |
| Intervention – Vitruvian plot | -10.02739032 | -3.4693512 |
| Sex - I do not know / prefer not to say | -3.17526043 | 38.1492069 |
| Sex – male | 0.02565479 | 5.6863116 |
| Ethnicity - Arab | -41.45939299 | 7.1162781 |
| Ethnicity - Asian/Asian British | -6.43711363 | 8.0434412 |
| Ethnicity - Black/African/Caribbean/Black British | 6.57573191 | 36.5948559 |
| Ethnicity - Chinese | -25.01694152 | 9.2362207 |
| Ethnicity - Mixed/Multiple ethnic groups | -0.73370836 | 19.5607994 |
| Ethnicity - Other | -7.63375162 | 15.0881424 |
| Age | -0.34018237 | -0.1513704 |
| NVS – total score | -3.00572602 | -0.6638898 |
| Education - Higher or secondary or further education (A-levels, BTEC, etc.) | -4.65823199 | 5.0214926 |
| Education - College or university or equivalent | -4.94309632 | 3.9574742 |
| Education - Post-graduate degree or equivalent | -2.84104222 | 6.5682069 |
| Education - I do not know / prefer not to say | -26.54196518 | 10.6330064 |
| Familiarity - I do not know / prefer not to say | -3.58281717 | 14.7863304 |
| Familiarity - Yes | -4.02379495 | 3.3039334 |

### 6.1.5 Support sub-scale – analysis

| Variable | Estimate | Std. Error | t value | Pr(>\|t\|) |
| --- | --- | --- | --- | --- |
| (Intercept) | 40.7524 | 4.2981 | 9.481 | < 2e-16 *** |
| Intervention - Kilim | -10.014 | 1.635 | -6.125 | 1.13e-09 *** |
| Intervention – Vitruvian plot | -12.0012 | 1.6465 | -7.289 | 4.78e-13 *** |
| Sex - I do not know / prefer not to say | 15.4377 | 10.375 | 1.488 | 0.136946 |
| Sex – male | 0.1345 | 1.4212 | 0.095 | 0.924598 |
| Ethnicity - Arab | -14.7725 | 12.1956 | -1.211 | 0.225948 |
| Ethnicity - Asian/Asian British | 1.4765 | 3.6355 | 0.406 | 0.684697 |
| Ethnicity - Black/African/Caribbean/Black British | 15.791 | 7.5367 | 2.095 | 0.036301 * |
| Ethnicity - Chinese | -6.2198 | 8.5997 | -0.723 | 0.469620 |
| Ethnicity - Mixed/Multiple ethnic groups | 7.2456 | 5.0952 | 1.422 | 0.155196 |
| Ethnicity - Other | 1.8568 | 5.7046 | 0.325 | 0.744844 |
| Age | -0.1388 | 0.0474 | -2.928 | 0.003458 ** |
| NVS – total score | -0.9446 | 0.5879 | -1.607 | 0.108327 |
| Education - Higher or secondary or further education (A-levels, BTEC, etc.) | 4.0329 | 2.4302 | 1.659 | 0.097205 . |
| Education - College or university or equivalent | 4.1166 | 2.2346 | 1.842 | 0.065618 . |
| Education - Post-graduate degree or equivalent | 8.2441 | 2.3623 | 3.49 | 0.000496 *** |
| Education - I do not know / prefer not to say | 6.6945 | 9.3333 | 0.717 | 0.473304 |
| Familiarity - I do not know / prefer not to say | 6.6459 | 4.6118 | 1.441 | 0.149755 |
| Familiarity - Yes | 3.1326 | 1.8397 | 1.703 | 0.088797 . |

```
---
Signif. codes:  0 ‘***’ 0.001 ‘**’ 0.01 ‘*’ 0.05 ‘.’ 0.1 ‘ ’ 1

Residual standard error: 27 on 1683 degrees of freedom
Multiple R-squared:  0.05954,	Adjusted R-squared:  0.04948
F-statistic: 5.919 on 18 and 1683 DF,  p-value: 4.447e-14
```

| Variable | 2.5 % | 97.5 % |
| --- | --- | --- |
| (Intercept) | 32.3221984 | 49.18268662 |
| Intervention - Kilim | -13.220902 | -6.80705759 |
| Intervention – Vitruvian plot | -15.2305602 | -8.77183046 |
| Sex - I do not know / prefer not to say | -4.9115916 | 35.78709301 |
| Sex – male | -2.6529419 | 2.92199466 |
| Ethnicity - Arab | -38.6925792 | 9.14750315 |
| Ethnicity - Asian/Asian British | -5.6541304 | 8.60714316 |
| Ethnicity - Black/African/Caribbean/Black British | 1.0087357 | 30.57327547 |
| Ethnicity - Chinese | -23.0870631 | 10.6473983 |
| Ethnicity - Mixed/Multiple ethnic groups | -2.7479543 | 17.23923063 |
| Ethnicity - Other | -9.3320605 | 13.04575247 |
| Age | -0.2317714 | -0.04581861 |
| NVS – total score | -2.0977919 | 0.20858157 |
| Education - Higher or secondary or further education (A-levels, BTEC, etc.) | -0.7336679 | 8.79947514 |
| Education - College or university or equivalent | -0.266249 | 8.49953883 |
| Education - Post-graduate degree or equivalent | 3.6106937 | 12.87745718 |
| Education - I do not know / prefer not to say | -11.6115086 | 25.00051673 |
| Familiarity - I do not know / prefer not to say | -2.3996164 | 15.6913644 |
| Familiarity - Yes | -0.4757674 | 6.74099603 |

## 6.2 Information comprehension (secondary outcome)

After participants were presented with a short clinical scenario, they were asked to indicate which medication they would opt for. One answer has been a-priori defined as correct during trial development and was tested with five members of the public. Comprehension questions have been previously used to evaluate the comprehension of data and information in decision aids (Schwartz et al. 2007; Schwartz et al. 2009). Below are two illustrative examples of comprehension questions:

- ***“Imagine you had bothersome heartburn. If you could take [either of the 2 drugs] for free, which drug would you rather take: [drug 1], [drug 2], neither drug?”.***

[drug 1] was considered the correct answer because it had substantially greater benefits than and similar side effects to [drug 2] (Schwartz et al. 2009, modified).

- ***Mrs Jones is mainly concerned about [adverse event 1] and [adverse event 2], while [adverse event 3] does not bother her at all. Which drug should Mrs Jones take if she can access [drug 1], [drug 2] and [drug 3] for free?”***

[drug 2] is considered the correct answer because it had lower event rates than [adverse event 1] and [adverse event 2] compared to [drug 3]. [drug 1] performed similarly to [drug 2] but was associated with a higher event rate of dropping out from the treatment and reduced efficacy (Schwartz et al. 2007, modified).

**References**

- Schwartz LM, Woloshin S, Welch HG. The drug facts box: providing consumers with simple tabular data on drug benefit and harm. Med Decis Making. 2007 Sep-Oct;27(5):655-62. doi: 10.1177/0272989X07306786. Epub 2007 Sep 14. PMID: 17873258.
- Schwartz LM, Woloshin S, Welch HG. Using a drug facts box to communicate drug benefits and harms: two randomized trials. Ann Intern Med. 2009 Apr 21;150(8):516-27. doi: 10.7326/0003-4819-150-8-200904210-00106. Epub 2009 Feb 16. PMID: 19221371.

### 6.2.1 Summary – analysis

| Variable | Estimate | Std. Error | z value | Pr(>\|z\|) |
| --- | --- | --- | --- | --- |
| (Intercept) | -1.661957 | 0.354998 | -4.682 | 2.85e-06 *** |
| Intervention - Kilim | 0.613612 | 0.134525 | 4.561 | 5.08e-06 *** |
| Intervention – Vitruvian plot | 1.243413 | 0.135399 | 9.183 | < 2e-16 *** |
| Sex - I do not know / prefer not to say | -0.292728 | 0.879914 | -0.333 | 0.73938 |
| Sex – male | 0.270978 | 0.112269 | 2.414 | 0.01579 * |
| Ethnicity - Arab | -0.363804 | 0.952536 | -0.382 | 0.70251 |
| Ethnicity - Asian/Asian British | -0.160972 | 0.287478 | -0.56 | 0.57552 |
| Ethnicity - Black/African/Caribbean/Black British | -0.785091 | 0.697647 | -1.125 | 0.26044 |
| Ethnicity - Chinese | 0.147096 | 0.666351 | 0.221 | 0.82529 |
| Ethnicity - Mixed/Multiple ethnic groups | -0.044981 | 0.402702 | -0.112 | 0.91106 |
| Ethnicity - Other | 0.269518 | 0.46575 | 0.579 | 0.56281 |
| Age | -0.016891 | 0.003784 | -4.464 | 8.05e-06 *** |
| NVS – total score | 0.24875 | 0.050602 | 4.916 | 8.84e-07 *** |
| Education - Higher or secondary or further education (A-levels, BTEC, etc.) | 0.206678 | 0.197788 | 1.045 | 0.29605 |
| Education - College or university or equivalent | 0.297734 | 0.182416 | 1.632 | 0.10264 |
| Education - Post-graduate degree or equivalent | 0.605054 | 0.191052 | 3.167 | 0.00154 ** |
| Education - I do not know / prefer not to say | -0.269628 | 0.855818 | -0.315 | 0.75272 |
| Familiarity - I do not know / prefer not to say | -0.639353 | 0.400457 | -1.597 | 0.11036 |
| Familiarity - Yes | -0.31299 | 0.148249 | -2.111 | 0.03475 * |

```
Signif. codes:  0 ‘***’ 0.001 ‘**’ 0.01 ‘*’ 0.05 ‘.’ 0.1 ‘ ’ 1

(Dispersion parameter for binomial family taken to be 1)

    Null deviance: 2289.5  on 1701  degrees of freedom
Residual deviance: 2132.1  on 1683  degrees of freedom
AIC: 2170.1

Number of Fisher Scoring iterations: 4
```

|  | OR | 2.5 % | 97.5 % |
| --- | --- | --- | --- |
| Variable | 0.19 | 0.09 | 0.38 |
| (Intercept) | 1.85 | 1.42 | 2.41 |
| Intervention - Kilim | 3.47 | 2.66 | 4.53 |
| Intervention – Vitruvian plot | 1.88 | 1.48 | 2.38 |
| Sex - I do not know / prefer not to say | 0.75 | 0.10 | 3.82 |
| Sex – male | 1.32 | 1.05 | 1.63 |
| Ethnicity - Arab | 0.70 | 0.09 | 4.51 |
| Ethnicity - Asian/Asian British | 0.85 | 0.48 | 1.49 |
| Ethnicity - Black/African/Caribbean/Black British | 0.46 | 0.10 | 1.61 |
| Ethnicity - Chinese | 1.16 | 0.29 | 4.22 |
| Ethnicity - Mixed/Multiple ethnic groups | 0.96 | 0.43 | 2.09 |
| Ethnicity - Other | 1.31 | 0.52 | 3.30 |
| Age | 0.98 | 0.98 | 0.99 |
| NVS – total score | 1.28 | 1.16 | 1.42 |
| Education - Higher or secondary or further education (A-levels, BTEC, etc.) | 1.23 | 0.84 | 1.82 |
| Education - College or university or equivalent | 1.35 | 0.95 | 1.93 |
| Education - Post-graduate degree or equivalent | 1.83 | 1.26 | 2.67 |
| Education - I do not know / prefer not to say | 0.76 | 0.11 | 3.59 |
| Familiarity - I do not know / prefer not to say | 0.53 | 0.23 | 1.12 |
| Familiarity - Yes | 0.73 | 0.55 | 0.98 |

**Green: odds ratio (OR) and lower 95% confidence interval (LCI) both > 1, improving information comprehension.**

**Red: OR and upper 95% confidence interval (UCI) both < 1, worsening information comprehension.**

## 6.3 Decision Self-Efficacy scale (secondary outcome)

The Decision Self-Efficacy (DSE) scale measures self-confidence and belief in one’s ability to make a decision and participation in shared decision-making (O’Connor 1995). It has been previously used to evaluate PDAs (Hopkin et al. 2019). The scale has 11 items, rated along a 5-point scale ranging from “not at all confident” to “very confident”, and the combined scores are converted to a 0 to 100 scale, with higher scores indicating higher self-efficacy. The scale has been validated previously and had an acceptable alpha score of 0.82 (O’Connor 1995).

**References**

- Hopkin G, Au A, Collier VJ, Yudkin JS, Basu S, Naci H. Combining Multiple Treatment Comparisons with Personalized Patient Preferences: A Randomized Trial of an Interactive Platform for Statin Treatment Selection. Med Decis Making. 2019 Apr;39(3):264-277. doi: 10.1177/0272989X19835239. Epub 2019 Mar 15. PMID: 30873906.
- O’Connor AM. User Manual - Decisional Self-Efficacy Scale [document on the Internet]. Ottawa: Ottawa Hospital Research Institute; 1995 [modified 2002].

### 6.3.1 Summary (total score) – analysis

| Variable | Estimate | Std. Error | t value | Pr(>\|t\|) |
| --- | --- | --- | --- | --- |
| (Intercept) | 48.80483 | 3.73473 | 13.068 | < 2e-16 *** |
| Intervention - Kilim | 7.77584 | 1.42072 | 5.473 | 5.09e-08 *** |
| Intervention – Vitruvian plot | 7.37349 | 1.43066 | 5.154 | 2.85e-07 *** |
| Sex - I do not know / prefer not to say | -9.20514 | 9.01507 | -1.021 | 0.307362 |
| Sex – male | 2.52438 | 1.23489 | 2.044 | 0.041088 * |
| Ethnicity - Arab | 5.83022 | 10.59694 | 0.55 | 0.582269 |
| Ethnicity - Asian/Asian British | -1.28751 | 3.15898 | -0.408 | 0.683639 |
| Ethnicity - Black/African/Caribbean/Black British | -23.39242 | 6.54877 | -3.572 | 0.000364 *** |
| Ethnicity - Chinese | 15.34944 | 7.47244 | 2.054 | 0.040116 * |
| Ethnicity - Mixed/Multiple ethnic groups | -6.26246 | 4.42731 | -1.415 | 0.157399 |
| Ethnicity - Other | -0.44009 | 4.95686 | -0.089 | 0.929264 |
| Age | 0.17394 | 0.04119 | 4.223 | 2.54e-05 *** |
| NVS – total score | 2.10784 | 0.51088 | 4.126 | 3.87e-05 *** |
| Education - Higher or secondary or further education (A-levels, BTEC, etc.) | 1.20424 | 2.11166 | 0.57 | 0.568564 |
| Education - College or university or equivalent | 2.31819 | 1.94169 | 1.194 | 0.232684 |
| Education - Post-graduate degree or equivalent | 1.68173 | 2.05266 | 0.819 | 0.412734 |
| Education - I do not know / prefer not to say | -9.61045 | 8.10984 | -1.185 | 0.236171 |
| Familiarity - I do not know / prefer not to say | -13.67858 | 4.00729 | -3.413 | 0.000657 *** |
| Familiarity - Yes | -6.2942 | 1.59857 | -3.937 | 8.57e-05 *** |

```
Signif. codes:  0 ‘***’ 0.001 ‘**’ 0.01 ‘*’ 0.05 ‘.’ 0.1 ‘ ’ 1

Residual standard error: 23.46 on 1683 degrees of freedom
Multiple R-squared:  0.08863,	Adjusted R-squared:  0.07888
F-statistic: 9.092 on 18 and 1683 DF,  p-value: < 2.2e-16
```

| Variable | 2.5 % | 97.5 % |
| --- | --- | --- |
| (Intercept) | 41.47962765 | 56.1300232 |
| Intervention - Kilim | 4.98928428 | 10.5623944 |
| Intervention – Vitruvian plot | 4.56743162 | 10.1795434 |
| Sex - I do not know / prefer not to say | -26.88706273 | 8.4767902 |
| Sex – male | 0.10229805 | 4.9464653 |
| Ethnicity - Arab | -14.95435115 | 26.6147966 |
| Ethnicity - Asian/Asian British | -7.48345785 | 4.9084311 |
| Ethnicity - Black/African/Caribbean/Black British | -36.23701128 | -10.5478263 |
| Ethnicity - Chinese | 0.69318116 | 30.0056894 |
| Ethnicity - Mixed/Multiple ethnic groups | -14.94608024 | 2.4211608 |
| Ethnicity - Other | -10.1623445 | 9.2821583 |
| Age | 0.09315141 | 0.2547293 |
| NVS – total score | 1.10581138 | 3.1098627 |
| Education - Higher or secondary or further education (A-levels, BTEC, etc.) | -2.93752328 | 5.3460041 |
| Education - College or university or equivalent | -1.49019216 | 6.1265658 |
| Education - Post-graduate degree or equivalent | -2.34430053 | 5.7077646 |
| Education - I do not know / prefer not to say | -25.51688789 | 6.2959899 |
| Familiarity - I do not know / prefer not to say | -21.53837374 | -5.8187801 |
| Familiarity - Yes | -9.42958742 | -3.1588059 |

## 6.4 Preparation for Decision Making scale (secondary outcome)

The Preparation for Decision Making (PDM) scale measures a user’s perception of how useful a decision support tool is in preparing them to talk to a health professional about a health decision (Graham & O’Connor 1995), and it has been previously used to evaluate PDAs (Hopkin et al. 2019). The original scale has 10 items, but a single item (‘‘Help you recognise that a decision needs to be made?’’) was omitted as it was considered not relevant to this study, as done by Hopkin and colleagues. Items are rated along a 5-point scale ranging from “not at all” to “a great deal”, and the combined scores are converted to a 0 to 100 scale, with higher scores indicating a higher perceived level of preparation. The scale has been validated previously and had an acceptable alpha score of 0.90 (Bennett et al. 2010).

**References**

- Bennett C, Graham ID, Kristjansson E, Kearing SA, Clay KF, O'Connor AM. Validation of a preparation for decision making scale. Patient Educ Couns. 2010 Jan;78(1):130-3. doi: 10.1016/j.pec.2009.05.012. Epub 2009 Jun 26. PMID: 19560303.
- Graham ID, O’Connor AM. User Manual - Preparation for Decision Making Scale [document on the Internet]. Ottawa: Ottawa Hospital Research Institute; 1995 [updated 2010].
- Hopkin G, Au A, Collier VJ, Yudkin JS, Basu S, Naci H. Combining Multiple Treatment Comparisons with Personalized Patient Preferences: A Randomized Trial of an Interactive Platform for Statin Treatment Selection. Med Decis Making. 2019 Apr;39(3):264-277. doi: 10.1177/0272989X19835239. Epub 2019 Mar 15. PMID: 30873906.

### 6.4.1 Summary (total score) – analysis

| Variable | Estimate | Std. Error | t value | Pr(>\|t\|) |
| --- | --- | --- | --- | --- |
| (Intercept) | 48.86901 | 4.0107 | 12.185 | < 2e-16 *** |
| Intervention - Kilim | 12.29024 | 1.5257 | 8.055 | 1.48e-15 *** |
| Intervention – Vitruvian plot | 8.71637 | 1.53638 | 5.673 | 1.65e-08 *** |
| Sex - I do not know / prefer not to say | -5.88592 | 9.68123 | -0.608 | 0.5433 |
| Sex – male | -0.33213 | 1.32614 | -0.25 | 0.8023 |
| Ethnicity - Arab | 4.50424 | 11.38 | 0.396 | 0.6923 |
| Ethnicity - Asian/Asian British | -2.28727 | 3.39241 | -0.674 | 0.5003 |
| Ethnicity - Black/African/Caribbean/Black British | -5.18644 | 7.03269 | -0.737 | 0.4609 |
| Ethnicity - Chinese | 10.94446 | 8.02461 | 1.364 | 0.1728 |
| Ethnicity - Mixed/Multiple ethnic groups | -5.45671 | 4.75447 | -1.148 | 0.2513 |
| Ethnicity - Other | 1.30567 | 5.32314 | 0.245 | 0.8063 |
| Age | 0.19982 | 0.04423 | 4.517 | 6.70e-06 *** |
| NVS – total score | 1.04743 | 0.54863 | 1.909 | 0.0564 . |
| Education - Higher or secondary or further education (A-levels, BTEC, etc.) | -2.86336 | 2.2677 | -1.263 | 0.2069 |
| Education - College or university or equivalent | -2.5318 | 2.08517 | -1.214 | 0.2248 |
| Education - Post-graduate degree or equivalent | -4.39513 | 2.20434 | -1.994 | 0.0463 * |
| Education - I do not know / prefer not to say | -12.83281 | 8.70912 | -1.473 | 0.1408 |
| Familiarity - I do not know / prefer not to say | -6.81311 | 4.30341 | -1.583 | 0.1136 |
| Familiarity - Yes | -1.36029 | 1.71669 | -0.792 | 0.4282 |

```
---
Signif. codes:  0 ‘***’ 0.001 ‘**’ 0.01 ‘*’ 0.05 ‘.’ 0.1 ‘ ’ 1

Residual standard error: 25.2 on 1683 degrees of freedom
Multiple R-squared:  0.06552,	Adjusted R-squared:  0.05553
F-statistic: 6.556 on 18 and 1683 DF,  p-value: 4.373e-16
```

| Variable | 2.5 % | 97.5 % |
| --- | --- | --- |
| (Intercept) | 41.00251781 | 56.73549659 |
| Intervention - Kilim | 9.2977767 | 15.28270888 |
| Intervention – Vitruvian plot | 5.7029663 | 11.72978214 |
| Sex - I do not know / prefer not to say | -24.87444496 | 13.10260127 |
| Sex – male | -2.93319566 | 2.26892884 |
| Ethnicity - Arab | -17.81619588 | 26.8246822 |
| Ethnicity - Asian/Asian British | -8.94105882 | 4.36652229 |
| Ethnicity - Black/African/Caribbean/Black British | -18.98017373 | 8.60730006 |
| Ethnicity - Chinese | -4.79480786 | 26.68373279 |
| Ethnicity - Mixed/Multiple ethnic groups | -14.78200458 | 3.86857965 |
| Ethnicity - Other | -9.13499891 | 11.74634515 |
| Age | 0.11305673 | 0.28657428 |
| NVS – total score | -0.02863557 | 2.12350405 |
| Education - Higher or secondary or further education (A-levels, BTEC, etc.) | -7.31117281 | 1.58446151 |
| Education - College or university or equivalent | -6.62159448 | 1.55799984 |
| Education - Post-graduate degree or equivalent | -8.71866605 | -0.07159778 |
| Education - I do not know / prefer not to say | -29.91464863 | 4.24902504 |
| Familiarity - I do not know / prefer not to say | -15.25370391 | 1.6274808 |
| Familiarity - Yes | -4.7273643 | 2.00679333 |

## 6.5 Time to completion (secondary outcome)

We will measure the overall individual time to completion. This outcome measures the time each participant requires to familiarise themselves with the intervention (starting time: administration of the intervention) and complete the questionnaires (ending time: completion of questionnaires), expressed in minutes. The overall time spent was considered a proxy measure of feasibility.

We repeated the analysis after removing the outliers, defined as participants who required more than 60 minutes.

### 6.5.1 Time to completion (secondary outcome) – without outliers

Summary of findings as a reference:

| Variable | Estimate | Std. Error | t value | Pr(>\|t\|) |
| --- | --- | --- | --- | --- |
| (Intercept) | 54.479 | 1.667 | 32.679 | < 2e-16 *** |
| Kilim | -16.365 | 2.263 | -7.233 | 7.29e-13 *** |
| Vitruvian plot | -19.373 | 2.268 | -8.541 | < 2e-16 *** |

```
---
Signif. codes:  0 ‘***’ 0.001 ‘**’ 0.01 ‘*’ 0.05 ‘.’ 0.1 ‘ ’ 1

Residual standard error: 36.52 on 1611 degrees of freedom
Multiple R-squared:  0.04876,	Adjusted R-squared:  0.04757
F-statistic: 41.29 on 2 and 1611 DF,  p-value: < 2.2e-16
```

| Variable | **2.5 %** | **97.5 %** |
| --- | --- | --- |
| (Intercept) | 51.20926 | 57.74907 |
| Kilim | -20.80319 | -11.92708 |
| Vitruvian plot | -23.82162 | -14.92395 |

Kilim plot as a reference:

| Variable | Estimate | Std. Error | t value | Pr(>\|t\|) |
| --- | --- | --- | --- | --- |
| (Intercept) | 38.114 | 1.530 | 24.914 | < 2e-16 *** |
| Kilim | 16.365 | 2.263 | 7.233 | 7.29e-13 *** |
| Vitruvian plot | -3.008 | 2.169 | -1.386 | 0.166 |

```
Signif. codes:  0 ‘***’ 0.001 ‘**’ 0.01 ‘*’ 0.05 ‘.’ 0.1 ‘ ’ 1

Residual standard error: 36.52 on 1611 degrees of freedom
Multiple R-squared:  0.04876,	Adjusted R-squared:  0.04757
F-statistic: 41.29 on 2 and 1611 DF,  p-value: < 2.2e-16
```

| Variable | 2.5 % | 97.5 % |
| --- | --- | --- |
| (Intercept) | 35.113362 | 41.114708 |
| Kilim | 11.927076 | 20.803187 |
| Vitruvian plot | -7.262516 | 1.247212 |

### 6.5.2 Time to completion (secondary outcome) – with outliers

Summary of findings as a reference:

```
Call:
lm(formula = dcs.uncertainty ~ intervention, data = df)

Residuals:
    Min      1Q  Median      3Q     Max
-54.191 -35.177  -4.191  20.809  64.823
```

| Variable | Estimate | Std. Error | t value | Pr(>\|t\|) | Significance |
| --- | --- | --- | --- | --- | --- |
| (Intercept) | 54.191 | 1.62 | 33.446 | < 2e-16 | *** |
| Kilim | -16.297 | 2.198 | -7.414 | 1.93E-13 | *** |
| Vitruvian plot | -19.014 | 2.208 | -8.613 | < 2e-16 | *** |

```
Signif. codes:  0 ‘***’ 0.001 ‘**’ 0.01 ‘*’ 0.05 ‘.’ 0.1 ‘ ’ 1

Residual standard error: 36.48 on 1699 degrees of freedom
Multiple R-squared:  0.0476,	Adjusted R-squared:  0.04647
F-statistic: 42.45 on 2 and 1699 DF,  p-value: < 2.2e-16
```

| Variable | 2.5% | 97.5% |
| --- | --- | --- |
| (Intercept) | 51.01338 | 57.36926 |
| Kilim | -20.60916 | -11.98576 |
| Vitruvian plot | -23.34391 | -14.68400 |

Kilim plot as a reference:

```
Call:
lm(formula = dcs.uncertainty ~ intervention, data = df)

Residuals:
    Min      1Q  Median      3Q     Max
-54.191 -35.177  -4.191  20.809  64.823

Coefficients:
```

| Variable | Estimate | Std. Error | t value | Pr(>\|t\|) | Significance |
| --- | --- | --- | --- | --- | --- |
| (Intercept) | 37.894 | 1.486 | 25.506 | < 2e-16 | *** |
| Kilim | 16.297 | 2.198 | 7.414 | 1.93E-13 | *** |
| Vitruvian plot | -2.716 | 2.111 | -1.287 | 0.198 |  |

```
---
Signif. codes:  0 ‘***’ 0.001 ‘**’ 0.01 ‘*’ 0.05 ‘.’ 0.1 ‘ ’ 1

Residual standard error: 36.48 on 1699 degrees of freedom
Multiple R-squared:  0.0476,	Adjusted R-squared:  0.04647
F-statistic: 42.45 on 2 and 1699 DF,  p-value: < 2.2e-16
```

| Variable | 2.5% | 97.5% |
| --- | --- | --- |
| (Intercept) | 34.97985 | 40.807873 |
| Kilim | 11.98576 | 20.609157 |
| Vitruvian plot | -6.85663 | 1.423631 |

## 6.6 Choice with participants’ own set of preferences (additional analysis)

```
Call:
glm(formula = as.factor(choice) ~ intervention + sex + ethnicity +
    age + nvs.total + education2 + familiarity1, family = binomial(link = "logit"),
    data = df)

Coefficients:
```

| Variable | Estimate | Std. Error | z value | Pr(>\|z\|) | Significance |
| --- | --- | --- | --- | --- | --- |
| (Intercept) | 0.9705 | 0.6029 | 1.61 | 0.1075 |  |
| Intervention - Kilim | 1.446 | 0.2525 | 5.726 | 1.03E-08 | *** |
| Intervention – Vitruvian plot | 1.469 | 0.2528 | 5.813 | 6.14E-09 | *** |
| Sex - I do not know / prefer not to say | 0.1648 | 1.422 | 0.116 | 0.9077 |  |
| Sex – male | 26.03% | 22.25% | 1.17 | 0.2421 |  |
| Ethnicity - Arab | 13.67 | 1027 | 0.013 | 0.9894 |  |
| Ethnicity - Asian/Asian British | -0.4558 | 0.4924 | -0.926 | 0.3546 |  |
| Ethnicity - Black/African/Caribbean/Black British | 0.02033 | 1.066 | 0.019 | 0.9848 |  |
| Ethnicity - Chinese | 14.24 | 726.1 | 0.02 | 0.9844 |  |
| Ethnicity - Mixed/Multiple ethnic groups | -0.08009 | 0.7616 | -0.105 | 0.9162 |  |
| Ethnicity - Other | 1.075 | 1.128 | 0.954 | 0.3403 |  |
| Age | -0.01185 | 0.007568 | -1.566 | 0.1174 |  |
| NVS – total score | 0.317 | 0.07336 | 4.322 | 1.55E-05 | *** |
| Education - Higher or secondary or further education (A-levels, BTEC, etc.) | 0.5229 | 0.3791 | 1.379 | 0.1678 |  |
| Education - College or university or equivalent | -0.03198 | 0.3205 | -0.1 | 0.9205 |  |
| Education - Post-graduate degree or equivalent | -0.003237 | 0.343 | -0.009 | 0.9925 |  |
| Education - I do not know / prefer not to say | -1.708 | 0.8449 | -2.022 | 0.0432 | * |
| Familiarity - I do not know / prefer not to say | -1.024 | 0.4964 | -2.064 | 0.0391 | * |
| Familiarity - Yes | 0.06074 | 0.2866 | 0.212 | 0.8321 |  |

```
---
Signif. codes:  0 ‘***’ 0.001 ‘**’ 0.01 ‘*’ 0.05 ‘.’ 0.1 ‘ ’ 1

(Dispersion parameter for binomial family taken to be 1)

    Null deviance: 857.48  on 1701  degrees of freedom
Residual deviance: 764.38  on 1683  degrees of freedom
AIC: 802.38

Number of Fisher Scoring iterations: 15
```

## 6.7 Drop-out (additional analysis)

```
Call:
glm(formula = as.factor(drop) ~ intervention, family = binomial(link = "logit"),
    data = df.drop)

Coefficients:
```

| Variable | Estimate | Std. Error | z value | Pr(>\|z\|) | Significance |
| --- | --- | --- | --- | --- | --- |
| (Intercept) | -1.48194 | 0.09464 | -15.658 | < 2e-16 | *** |
| Kilim | 0.53154 | 0.12662 | 4.198 | 2.69E-05 | *** |
| Vitruvian plot | 0.06127 | 0.13282 | 0.461 | 6.45E-01 |  |

```
---
Signif. codes:  0 ‘***’ 0.001 ‘**’ 0.01 ‘*’ 0.05 ‘.’ 0.1 ‘ ’ 1

(Dispersion parameter for binomial family taken to be 1)

    Null deviance: 2287.2  on 2177  degrees of freedom
Residual deviance: 2265.5  on 2175  degrees of freedom
AIC: 2271.5

Number of Fisher Scoring iterations: 4
```

## 6.8 Overview of prognostic factors

|  | **Decisional Conflict Scale** | | | | | **Information comprehension (odds ratio)** | **Decision Self-Efficacy** | **Preparation to Decision-Making** |
| --- | --- | --- | --- | --- | --- | --- | --- | --- |
|  | **summary** | **knowledge** | **support** | **certainty** | **values clarity** |  |  |  |
| ***Sex assigned at birth (reference: female)*** | | | | | | | | |
| Male |  |  |  |  | 2.9 [0.0; 5.7] | 1.3 [1.1; 1.6] | 2.5 [0.1; 5.0] |  |
| ***Ethnicity (reference: white)*** | | | | | | | | |
| Black | 15.8 [3.7; 27.9] | 22.6 [9.0; 36.1] | 15.8 [1.0; 30.6] |  | 21.6 [6.6; 36.6] |  | -23.4 [-36.2; 10.6] |  |
| Chinese |  |  |  | -32.8 [-55.3; -10.5] |  |  | 15.4 [0.7; 30.0] |  |
| Mixed/multiple |  | 12.3 [3.1; 21.5] |  |  |  |  |  |  |
| ***Familiarity with the medical condition of interest (reference: no lived experience)*** | | | | | | | | |
| I do not know |  |  |  |  |  |  | -13.7 [-21.5; -5.8] |  |
| Yes | 3.0 [0.0; 5.9] | 5.2 [1.9; 8.5] |  |  |  | 0.7 [0.6; 0.98] | -6.3 [-9.4; -3.2] |  |
| ***Other (increasing values of row-defining prognostic factors)*** | | | | | | | | |
| Age | -0.2 [-0.3; -0.2] | -0.2 [-0.3; -0.1] | -0.1 [-0.2; -0.1] | -0.4 [-0.5; -0.3] | -0.2 [-0.3; -0.2] | 0.98 [0.98; 0.99] | 0.2 [0.1; 0.3] | 0.2 [0.1; 0.3] |
| Literacy and numeracy | -1.4 [-2.4; -0.5] | -2.3 [-3.4; -1.3] |  |  | -1.8 [-3.0; -0.7] | 1.3 [1.2; 1.4] | 2.1 [1.1; 3.1] |  |

♦ positive association (i.e. improved performance of the row-defining prognostic factors on the column-defining outcome)

♦ negative association (i.e. worsened performance of the row-defining prognostic factors on the column-defining outcome)

To explore potential prognostic factors associated with the considered outcomes, we conducted multivariable linear regression analyses (see supplementary sections 6.1-6.5) to account for the simultaneous influence of multiple covariates. All variables included in the model were pre-specified during the trial design phase. Selection was based on their perceived clinical importance and was done a priori, i.e. before data collection. This approach was chosen to avoid a posteriori, data-driven selection, and to ensure that the analysis remained hypothesis-driven.

# 7. ASSOCIATIONS/CAUSAL PATHWAYS BETWEEN OUTCOMES

## 7.1 Outcomes correlation

We repeated the analysis after removing the outliers, defined as participants who required more than 60 minutes.

### 7.1.1 Outcomes correlation (with outliers)

\# Correlation Matrix (auto-method)

| Parameter1 | Parameter2 | r | 95% CI | t(1700) | p | Significance |
| --- | --- | --- | --- | --- | --- | --- |
| dse.total | pdm.total | 0.63 | [0.60, 0.66] | 33.82 | < .001 | *** |
| dse.total | dcs.total | -0.6 | [-0.63, -0.57] | -3.11E+01 | < .001 | *** |
| dse.total | time | -0.000378 | [-0.05, 0.05] | -2.00E-02 | > .999 |  |
| pdm.total | dcs.total | -0.61 | [-0.64, -0.58] | -32.15 | < .001 | *** |
| pdm.total | time | 1.00% | [-0.04, 0.06] | 0.49 | > .999 |  |
| dcs.total | time | -0.03 | [-0.07, 0.02] | -1.12 | 0.794 |  |

```
p-value adjustment method: Holm (1979)
Observations: 1702

sof:
# Correlation Matrix (auto-method)
```

| Parameter1 | Parameter2 | r | 95% CI | t(505) | p | Significance |
| --- | --- | --- | --- | --- | --- | --- |
| dse.total | pdm.total | 0.65 | [0.59, 0.70] | 19.13 | < .001 | *** |
| dse.total | dcs.total | -0.59 | [-0.64, -0.53] | -1.63E+01 | < .001 | *** |
| dse.total | time | -0.04 | [-0.13, 0.05] | -8.70E-01 | > .999 |  |
| pdm.total | dcs.total | -0.62 | [-0.67, -0.57] | -17.95 | < .001 | *** |
| pdm.total | time | -0.81% | [-0.10, 0.08] | -0.18 | > .999 |  |
| dcs.total | time | -0.01 | [-0.10, 0.08] | -0.24 | > .999 |  |

```
p-value adjustment method: Holm (1979)
Observations: 507

kilim:
# Correlation Matrix (auto-method)
```

| Parameter1 | Parameter2 | r | 95% CI | t(601) | p | Significance |
| --- | --- | --- | --- | --- | --- | --- |
| dse.total | pdm.total | 0.62 | [0.57, 0.67] | 19.29 | < .001 | *** |
| dse.total | dcs.total | -0.6 | [-0.65, -0.55] | -1.84E+01 | < .001 | *** |
| dse.total | time | 0.04 | [-0.04, 0.12] | 9.20E-01 | 0.476 |  |
| pdm.total | dcs.total | -0.59 | [-0.64, -0.53] | -17.8 | < .001 | *** |
| pdm.total | time | 5.00% | [-0.03, 0.13] | 1.18 | 0.476 |  |
| dcs.total | time | -0.08 | [-0.15, 0.00] | -1.85 | 0.196 |  |

```
p-value adjustment method: Holm (1979)
Observations: 603

vplot:
# Correlation Matrix (auto-method)
```

| Parameter1 | Parameter2 | r | 95% CI | t(590) | p | Significance |
| --- | --- | --- | --- | --- | --- | --- |
| dse.total | pdm.total | 0.61 | [0.55, 0.65] | 18.46 | < .001 | *** |
| dse.total | dcs.total | -0.58 | [-0.63, -0.53] | -1.74E+01 | < .001 | *** |
| dse.total | time | -0.07 | [-0.15, 0.01] | -1.68E+00 | 0.174 |  |
| pdm.total | dcs.total | -0.59 | [-0.64, -0.54] | -17.8 | < .001 | *** |
| pdm.total | time | -7.00% | [-0.15, 0.01] | -1.75 | 0.174 |  |
| dcs.total | time | 0.08 | [0.00, 0.16] | 1.9 | 0.174 |  |

p-value adjustment method: Holm (1979)

Observations: 592

### 7.1.2 Outcomes correlation (without outliers)

\# Correlation Matrix (auto-method)

| Parameter1 | Parameter2 | r | 95% CI | t(1612) | p | Significance |
| --- | --- | --- | --- | --- | --- | --- |
| DSE, total | PDM, total | 0.64 | [0.60, 0.66] | 33.01 | < .001 | *** |
| DSE, total | DCS, total | -0.6 | [-0.63, -0.57] | -3.03E+01 | < .001 | *** |
| DSE, total | time | 0.08 | [0.04, 0.13] | 3.39E+00 | 0.002 | ** |
| PDM, total | DCS, total | -0.61 | [-0.64, -0.58] | -31.17 | < .001 | *** |
| PDM, total | time | 6.00% | [0.02, 0.11] | 2.6 | 0.019 | * |
| DCS, total | time | -0.03 | [-0.08, 0.02] | -1.1 | 0.27 |  |

```
p-value adjustment method: Holm (1979)
Observations: 1614

sof:
# Correlation Matrix (auto-method)
```

| Parameter1 | Parameter2 | r | 95% CI | t(478) | p | Significance |
| --- | --- | --- | --- | --- | --- | --- |
| DSE, total | PDM, total | 0.66 | [0.61, 0.71] | 19.19 | < .001 | *** |
| DSE, total | DCS, total | -0.6 | [-0.66, -0.54] | -1.65E+01 | < .001 | *** |
| DSE, total | time | 0.15 | [0.06, 0.24] | 3.39E+00 | 0.002 | ** |
| PDM, total | DCS, total | -0.63 | [-0.68, -0.57] | -17.54 | < .001 | *** |
| PDM, total | time | 20.00% | [0.11, 0.29] | 4.48 | < .001 | *** |
| DCS, total | time | -0.11 | [-0.20, -0.02] | -2.39 | 0.017 | * |

```
p-value adjustment method: Holm (1979)
Observations: 480

kilim:
# Correlation Matrix (auto-method)
```

| Parameter1 | Parameter2 | r | 95% CI | t(568) | p | Significance |
| --- | --- | --- | --- | --- | --- | --- |
| DSE, total | PDM, total | 0.62 | [0.56, 0.67] | 18.72 | < .001 | *** |
| DSE, total | DCS, total | -0.6 | [-0.65, -0.54] | -1.78E+01 | < .001 | *** |
| DSE, total | time | 0.1 | [0.01, 0.18] | 2.32E+00 | 0.062 |  |
| PDM, total | DCS, total | -0.59 | [-0.64, -0.54] | -17.49 | < .001 | *** |
| PDM, total | time | 4.00% | [-0.04, 0.12] | 0.98 | 0.655 |  |
| DCS, total | time | -0.01 | [-0.10, 0.07] | -0.34 | 0.73 |  |

```
p-value adjustment method: Holm (1979)
Observations: 570

vplot:
# Correlation Matrix (auto-method)
```

| Parameter1 | Parameter2 | r | 95% CI | t(562) | p | Significance |
| --- | --- | --- | --- | --- | --- | --- |
| DSE, total | PDM, total | 0.6 | [0.54, 0.65] | 17.65 | < .001 | *** |
| DSE, total | DCS, total | -0.57 | [-0.62, -0.51] | -1.65E+01 | < .001 | *** |
| DSE, total | time | 0.07 | [-0.01, 0.15] | 1.63E+00 | 0.313 |  |
| PDM, total | DCS, total | -0.58 | [-0.63, -0.52] | -16.83 | < .001 | *** |
| PDM, total | time | 4.00% | [-0.04, 0.12] | 0.94 | 0.697 |  |
| DCS, total | time | -0.03 | [-0.11, 0.05] | -0.74 | 0.697 |  |

p-value adjustment method: Holm (1979)

Observations: 564

Legend:

DCS = Decisional Conflict Scale low literacy

DSE = Decision Self-Efficacy

PDM = Preparation for Decision-Making

## 7.2 Information comprehension (association with other outcomes)

### 7.2.1 Decisional Conflict Scale low literacy (total score)

```
Call:
lm(formula = dcs.total ~ info.comprehension, data = df)

Residuals:
    Min      1Q  Median      3Q     Max
-25.850 -17.703  -7.703  14.150  77.297

Coefficients:
```

| Variable | Estimate | Std. Error | t value | Pr(>\|t\|) | Significance |
| --- | --- | --- | --- | --- | --- |
| (Intercept) | 25.8504 | 0.7222 | 35.795 | < 2e-16 | *** |
| Information comprehension, yes | -3.1479 | 1.1434 | -2.753 | 5.96E-03 | ** |

```
---
Signif. codes:  0 ‘***’ 0.001 ‘**’ 0.01 ‘*’ 0.05 ‘.’ 0.1 ‘ ’ 1

Residual standard error: 23.1 on 1700 degrees of freedom
Multiple R-squared:  0.004439,	Adjusted R-squared:  0.003853
F-statistic:  7.58 on 1 and 1700 DF,  p-value: 0.005965


                          2.5 %     97.5 %
(Intercept)           24.433978 27.2669021
info.comprehensionyes -5.390526 -0.9053461
```

### 7.2.2 Decision Self-Efficacy (total score)

```
Call:
lm(formula = dse.total ~ info.comprehension, data = df)

Residuals:
    Min      1Q  Median      3Q     Max
-74.759 -13.621   4.561  20.470  29.561

Coefficients:
```

| Variable | Estimate | Std. Error | t value | Pr(>\|t\|) | Significance |
| --- | --- | --- | --- | --- | --- |
| (Intercept) | 70.439 | 0.7617 | 92.482 | < 2e-16 | *** |
| Information comprehension, yes | 4.32 | 1.2059 | 3.582 | 3.50E-04 | *** |

```
---
Signif. codes:  0 ‘***’ 0.001 ‘**’ 0.01 ‘*’ 0.05 ‘.’ 0.1 ‘ ’ 1

Residual standard error: 24.36 on 1700 degrees of freedom
Multiple R-squared:  0.007493,	Adjusted R-squared:  0.006909
F-statistic: 12.83 on 1 and 1700 DF,  p-value: 0.00035


                          2.5 %    97.5 %
(Intercept)           68.945116 71.932872
info.comprehensionyes  1.954853  6.685167
```

### 7.2.3 Preparation for Decision-Making (total score)

```
Call:
lm(formula = pdm.total ~ info.comprehension, data = df)

Residuals:
    Min      1Q  Median      3Q     Max
-69.698 -14.964   5.302  21.147  35.036

Coefficients:
```

| Variable | Estimate | Std. Error | t value | Pr(>\|t\|) | Significance |
| --- | --- | --- | --- | --- | --- |
| (Intercept) | 64.9642 | 0.8076 | 80.446 | < 2e-16 | *** |
| Information comprehension, yes | 4.7339 | 1.2785 | 3.703 | 2.20E-04 | *** |

```
---
Signif. codes:  0 ‘***’ 0.001 ‘**’ 0.01 ‘*’ 0.05 ‘.’ 0.1 ‘ ’ 1

Residual standard error: 25.83 on 1700 degrees of freedom
Multiple R-squared:  0.008,	Adjusted R-squared:  0.007416
F-statistic: 13.71 on 1 and 1700 DF,  p-value: 0.0002202


                          2.5 %    97.5 %
(Intercept)           63.380252 66.548063
info.comprehensionyes  2.226236  7.241619
```

### 7.2.4 Time to completion (minutes)

```
Call:
lm(formula = time ~ info.comprehension, data = df)

Residuals:
   Min     1Q Median     3Q    Max
  -225   -208   -104    -97  43187

Coefficients:
```

| Variable | Estimate | Std. Error | t value | Pr(>\|t\|) | Significance |
| --- | --- | --- | --- | --- | --- |
| (Intercept) | 113.36 | 47.08 | 2.408 | 0.0161 | * |
| Information comprehension, yes | 113.48 | 74.53 | 1.523 | 1.28E-01 |  |

```
---
Signif. codes:  0 ‘***’ 0.001 ‘**’ 0.01 ‘*’ 0.05 ‘.’ 0.1 ‘ ’ 1

Residual standard error: 1506 on 1700 degrees of freedom
Multiple R-squared:  0.001362,	Adjusted R-squared:  0.0007744
F-statistic: 2.318 on 1 and 1700 DF,  p-value: 0.1281


                          2.5 %   97.5 %
(Intercept)            21.02910 205.6941
info.comprehensionyes -32.70417 259.6635
```

## 7.3 Information comprehension

```
Call:
glm(formula = as.factor(info.comprehension) ~ sex + ethnicity +
    age + nvs.total + education2 + familiarity1, family = binomial(link = "logit"),
    data = df)

Coefficients:
```

| Variable | Estimate | Std. Error | z value | Pr(>\|z\|) | Significance |
| --- | --- | --- | --- | --- | --- |
| (Intercept) | -1.071403 | 0.336159 | -3.187 | 0.001437 | ** |
| Sex - I do not know / prefer not to say | -0.419531 | 0.853036 | -0.492 | 6.23E-01 |  |
| Sex – male | 0.310418 | 0.109066 | 2.846 | 4.43E-03 | ** |
| Ethnicity - Arab | -0.233233 | 0.941134 | -0.248 | 0.804272 |  |
| Ethnicity - Asian/Asian British | -7.74% | 27.92% | -0.277 | 0.781691 |  |
| Ethnicity - Black/African/Caribbean/Black British | -0.741444 | 0.685244 | -1.082 | 0.279246 |  |
| Ethnicity - Chinese | -0.237682 | 0.658331 | -0.361 | 0.718072 |  |
| Ethnicity - Mixed/Multiple ethnic groups | -0.019128 | 0.391603 | -0.049 | 0.961042 |  |
| Ethnicity - Other | 0.341106 | 0.442661 | 0.771 | 0.440955 |  |
| Age | -0.013769 | 0.003651 | -3.772 | 0.000162 | *** |
| NVS – total score | 0.238026 | 0.049154 | 4.842 | 1.28E-06 | *** |
| Education - Higher or secondary or further education (A-levels, BTEC, etc.) | 0.153987 | 0.192907 | 0.798 | 0.42473 |  |
| Education - College or university or equivalent | 0.234049 | 0.177974 | 1.315 | 1.88E-01 |  |
| Education - Post-graduate degree or equivalent | 0.554157 | 0.186137 | 2.977 | 0.002909 | ** |
| Education - I do not know / prefer not to say | -0.428347 | 0.836232 | -0.512 | 0.608486 |  |
| Familiarity - I do not know / prefer not to say | -0.60561 | 0.389012 | -1.557 | 0.119521 |  |
| Familiarity - Yes | -0.236055 | 0.143985 | -1.639 | 0.10112 |  |

```
---
Signif. codes:  0 ‘***’ 0.001 ‘**’ 0.01 ‘*’ 0.05 ‘.’ 0.1 ‘ ’ 1

(Dispersion parameter for binomial family taken to be 1)

    Null deviance: 2289.5  on 1701  degrees of freedom
Residual deviance: 2222.5  on 1685  degrees of freedom
AIC: 2256.5

Number of Fisher Scoring iterations: 4
```

# 8 FURTHER DETAILS

## 8.1 Newest Vital Sign (United Kingdom version)

The NVS-UK is a measure scale of health literacy skills. It takes an average of 3 minutes to complete and can be administered by both clinical and non-clinical personnel. It has been validated against the Test of Functional Health Literacy in Adults (TOFHLA), the gold standard test of health literacy (Pearson’s r of 0.49, area under the ROC curve of 0.81) (Rowlands et al. 2013; Weiss et al. 2005).

The NVS is a quick, 6-question test that assesses health literacy (numeracy and literacy), scoring patients as having a high likelihood of limited literacy (0-1 correct), limited literacy (2-3 correct), or adequate literacy (4-6 correct).
